# Target-Genes Reveal Species and Genotypic Specificity of Anthocyanin Pigmentation in *Citrus* and Related Genera

**DOI:** 10.3390/genes11070807

**Published:** 2020-07-16

**Authors:** Chiara Catalano, Angelo Ciacciulli, Fabrizio Salonia, Maria Patrizia Russo, Paola Caruso, Marco Caruso, Giuseppe Russo, Gaetano Distefano, Concetta Licciardello

**Affiliations:** 1 CREA, Research Centre for Olive, Fruit and Citrus Crops, Corso Savoia 190, 95024 Acireale, Italy; chiara.catalano@phd.unict.it (C.C.); angelo.ciacciulli@crea.gov.it (A.C.); fabrizio.salonia@unict.it (F.S.); mariapatrizia.russo@crea.gov.it (M.P.R.); paola.caruso@crea.gov.it (P.C.); marco.caruso@crea.gov.it (M.C.); giuseppe.russo@crea.gov.it (G.R.); 2 Department of Agriculture, Food and Environment (Di3A), University of Catania, Via Valdisavoia 5, 95123 Catania, Italy; distefag@unict.it

**Keywords:** red color, *Citrus*, fruit, stamen, stigma, style, image analysis, qRT-PCR

## Abstract

Background: Anthocyanin pigmentation characterizes a number of tissues of *Citrus* and its relatives. The gain and loss of pigmentation is intriguing and is inherited variously among species. Methods: *Citrus* germplasm was used to investigate the anthocyanin pigmentation of tissues never before considered, including stamen, style and stigma, and of young leaves, petals, rind and flesh of 28 genotypes belonging to 14 species. *Citrus* genotypes encompassed citron, lemon, sweet orange, lime, and *Citrus* relatives included *Microcitrus*, *Murraya*, and *Severinia*. A relative qRT-PCR analysis was carried out on the structural and regulatory genes: phenylalanine ammonia-lyase (PAL), chalcone synthase (CHS), chalcone isomerase (CHI), flavanone 3′-hydroxylase (F3H), dihydroflavonol 4-reductase (DFR), anthocyanidin synthase (ANS), uridine diphosphate glucose flavonoid glucosyl-transferase (UFGT), glutathione S-transferase (GST), Ruby and Noemi. Image analysis and a genomic approach were employed to evaluate how the red pigmentation is inherited among tissues and species. Results: Pigmentation of young leaves and petals is specific to citron and its hybrids. Ruby controls the pigmentation of petals, but not of leaves. The red color of the rind and flesh is a trait that particularly characterizes a diversity of sweet oranges, citron hybrids and *Citrus* relatives. Color expression depends on external factors and also on developmental stage. The coloration of stamen and style is citron-specific, while a red stigma is exclusive to Moro orange and its hybrids. Conclusion: It is hypothesized that there is a relationship among *Citrus* species and genes controlling anthocyanin pigmentation.

## 1. Introduction

Sweet oranges, lemons, limes, grapefruits, and pummelos are all economically important species belonging to the *Citrus* genus. A recent study [1] described ten natural *Citrus* species, hypothesizing that they developed sometime from the late Miocene (10.4–10.5 million years ago) to the early Pliocene (5.3–5.4 million years ago). It is thought that in addition to mandarin (*Citrus reticulata*), pummelo (*Citrus maxima*), and citron (*Citrus medica*), which are defined as the ‘primary’ species from which most cultivated *Citrus* have originated, another two species are crucial to the origin of limes and calamondin, namely, *Citrus micrantha* (belonging to the subgenus Papeda) and *Fortunella japonica* (also known as kumquat) [2].

*Citrus* species and their relatives, such as *Microcitrus australasica* spp., *Severinia* spp., *Citrus latipes*, and *Citrus hystrix*, exhibit significant variability in anthocyanin pigmentation among their different tissues, especially in their leaves and petals. The accumulation of anthocyanins in the mature fruits is a particular feature that characterizes the flesh and the rind of ‘blood’ oranges (cultivars Moro, Tarocco, and Sanguinello) and occurs exclusively in the rind of ancient pummelos [3,4].

Anthocyanins are water-soluble pigments, and represent the largest class of the flavonoid family. They are responsible for the red to purple color of plant tissues. The different hues are the result of vacuolar pH, co-pigmentation with other polyphenols, and the presence of ionic complexes [5]. Anthocyanins are involved in a range of aspects of plant reproduction and in defense including in seed dispersal and protection against biotic and abiotic stresses [6,7]. Furthermore, fruits and vegetables rich in anthocyanins are highly sought after by consumers due to their visual attractiveness and to their nutraceutical properties—they are well known to protect the human body from cardiovascular diseases and several types of cancer, in addition to offering defense from damage caused by free radicals [8]. A slimming effect caused by the anthocyanins in Moro orange juice has been demonstrated in obese mice subjected to a high fat diet [9].

Environmental conditions play a crucial role in controlling anthocyanin pigmentation. In the various blood orange cultivars, the anthocyanin accumulation is cold-dependent. Storage conditions in the range 4 to 9 °C have been shown to increase the transcription levels of the associated biosynthetic and regulatory genes, as well as increasing the anthocyanin content [10,11,12,13]. Natural low temperatures, typical of winter, induce increases in the accumulations of anthocyanins in sweet oranges [14]. In *Citrus*, as in most other species, light also regulates pigmentation [15].

Two categories of genes are necessary for anthocyanin production: structural genes, encoding for enzymes directly involved in anthocyanin biosynthesis, and the complex WMBW made up of transcription factors (i.e., WRKY, MYB, bHLH, and WD-repeats), that control the tissue-specific expression of the structural genes. These have all been studied extensively [16]. The most important are: (1) phenylalanine ammonia-lyase (PAL), cinnamate 4-hydroxylase and 4-coumarate-CoA ligase, involved in the phenylpropanoid pathway; (2) early biosynthetic genes (EBGs), working in the first steps of the anthocyanin pathway and including chalcone synthase (CHS), chalcone isomerase (CHI) and flavanone 3′-hydroxylase (F3H); (3) late biosynthetic genes (LBGs), such as dihydroflavonol 4-reductase (DFR), anthocyanidin synthase (ANS), and uridine diphosphate glucose flavonoid glucosyl-transferase (UFGT). Lastly, glutathione S-transferase (GST) is responsible for the vacuolization of anthocyanins. It has been reported in some plant species that the EBGs are not expressed differently between pigmented and non-pigmented examples; this contrasts with the LBGs [17,18]. For the regulatory genes in *Citrus*, it has been demonstrated that the insertion of a Copia-like Long Terminal Repeat (LTR) retrotransposon, Tcs1, promotes the expression of Ruby, the MYB-like gene that, together with Noemi (bHLH-like) and a WD40 repeat, control the anthocyanin trait in the pigmented orange fruits [12,19]. In addition to Ruby, it has recently been reported that Ruby2 works as the activator responsible for anthocyanin accumulation in both modern and primitive *Citrus* [4]. More recently it was recognized in Noemi the gene that controls both the anthocyanin trait and the acidity trait in *Citrus* fruits [19]. Moreover, several MYB repressors have been shown to contribute to the negative regulation of anthocyanin biosynthesis [20]. Additionally, CsMYB3 working together with Ruby, has been reported to form a regulatory ‘activator-and-repressor’ loop, operating in *Citrus* and its wild relatives [21].

A number of studies have focused on tissue pigmentation of the sweet orange cultivars, especially that of the flesh [10,22,23,24,25,26]. However, no results have been reported for tissues other than the fruit, or in species outside the *Citrus* genus, where red and purple colors are present in the young leaves and flowers.

The present work employs transcriptional analysis to investigate the main structural genes (e.g., PAL, CHS, CHI, F3H, DFR, ANS, UFGT, and GST) and regulatory genes (e.g., Ruby and Noemi). Here, these are evaluated in a set of tissues including: young leaves, petals, stamens, styles, stigmas, flesh/juice, and rind. Some of these have not been evaluated previously in 28 genotypes belonging to 14 species (*C. medica*, *C. sinensis*, *C. limon*, *C. limonia*, *C. aurantifolia*, *C. latifolia*, *C. meyeri*, *C. celebica*, *C. latipes*, *C. hystrix*, *Microcitrus australasica*, *Murraya paniculata*, *Severina disticha*, and *S. buxifolia*). The genotypes were chosen based on the variability of pigmentation in a number of their tissues. This study aims to elucidate the phenotypic and genotypic variability of red–purple pigmentation. It suggests possible inheritance traits in each tissue across these species. The results of the study advance our understanding of the control of anthocyanin expression, and provides a foundation for further cultivar improvement and for the breeding of novel genotypes. Furthermore, it proposes new phenotypic markers, such as a purple stigma, that can be used to trace breeding materials.

## 2. Materials and Methods

### 2.1. Plant Material

**Figure 1:**
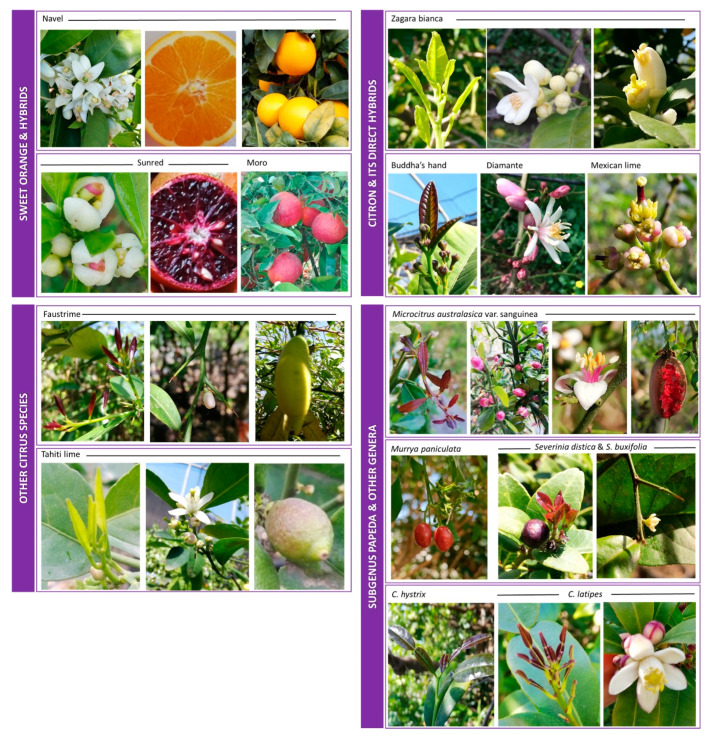
Variability in the levels of anthocyanins in the tissues of the accessions used in this study. These are combined into four groups as indicated in Table 1. For each group is presented a range of images of the most indicative accessions in terms of pigmentation in the young leaves, petals, stamens, styles, stigmas, flesh, and rind. Unpigmented examples are also shown for all tissues.

Plant materials were collected between November 2018 and April 2019 from the germplasm collection in Acireale and from the experimental orchard in Lentini of the Council for Agricultural Research and Economics-CREA (Table 1, Figure 1). Details on the geo-metereological conditions and soil compositions are reported in Figure S1. The tissues sampled for transcriptional analysis are: the young leaves, petals, stamens, styles, stigmas, flesh/juice, and rind. From one to three negative controls (unpigmented) for each tissue were also collected. All samples were stored at −80 °C pending RNA extraction.

The 28 accessions selected for our study belong to 14 species. They were gathered in four groups in consideration of their parentage: (1) sweet orange and its hybrids, (2) citron and its hybrids, (3) other *Citrus* species, and (4) subgenus Papeda and related genera. From one to three negative controls (no anthocyanin pigmentation) for each tissue were also selected, depending on the variability of the pigmentation and the availability of the tissues and genotypes for each group. The main traits and the origins of the accessions are described in Table 1.

### 2.2. Total RNA Extraction and Expression Analysis

Total RNA was extracted from: (1) 3 mL of filtered juice, (2) 2 g of rind and pulp (exclusively for *M. australasica* and *Faustrime*), and (3) 100 mg of young leaves, petals, stamens, styles, and stigmas, previously homogenized for 30 s at 30 rpm using the Tissuelyser II (Qiagen). One volume of extraction buffer (0.2 M TRIS pH 8.0, 0.2 M NaCl, 50 mM EDTA, 2% (w/v) SDS), one volume of phenol, and 0.02 volume of β-mercaptoethanol were added to the sample. After incubation at 50 °C for 5 min, samples were centrifuged at 4000 rpm at 4 °C for 15 min. Two cycles of centrifugation were carried out, adding to the upper aqueous phase one volume of chloroform:isoamyl alcohol (24:1, v/v). RNA was precipitated, adding one-half volume of 6 M LiCl to the upper phase at −20 °C overnight. After centrifugation at 8500 rpm for 40 min, the precipitated RNA was washed with 70% (v/v) ethanol and centrifuged at 7500 rpm for 20 min. The total RNA was resuspended in 50 μL of RNase-free water. The qualities and the quantities were evaluated using a Nanodrop 1000 spectrophotometer (Thermo Scientific) and by gel electrophoresis (agarose 0.8% in TAE 1×). The quality was considered optimal for values of 260/280 between 1.80 and 2.0.

DNAse treatment was carried out by adding to 40 μL of RNA 1× of RNaseOUT Recombinant Ribonuclease Inhibitor (Invitrogen), 0.1 M of DTT (Invitrogen), 5× Buffer, 1× of DNAse in a final volume of 50 μL. Samples were incubated at 37 °C for 30 min and purified using RNA Cleanup protocol (Qiagen), according to the manufacturer’s protocol. cDNA synthesis was carried out as previously described [12] using the High-Capacity cDNA Reverse Transcription Kit (Thermo Fisher) and specifically adding to 500 ng of RNA 1× RT Buffer, 4 mM of dNTPs, 1× RT Random Primers, 0.1× of MultiScribe Reverse Transcriptase, in a final volume of 20 μL. Thermal cycler conditions for cDNA synthesis were: 10 min at 25 °C, 37 °C for 120 min, and 85 °C for 5 s.

Relative quantification qRT-PCR was carried out using the ABI 7300 Real Time PCR System (Applied Biosystems). The PCR mixture (final volume, 15 μL) contained 7.5 μL Power SYBR Green PCR Master mix (Applied Biosystems), 0.1 mM of each gene-specific forward and reverse primer and 100 ng of the cDNA sample, according to the manufacturer’s protocol. The following standard thermal profile was used for all PCRs: 50 °C for 2 min, 95 °C for 10 min, 40 cycles of 95 °C for 15 s, and 60 °C for 1 min. Three technical replicates were assayed and a no-template negative control was routinely included in each plate. Data analyses were carried out using the standard curve method. For each gene of each tissue the calibrator was represented by the sample with the lowest ratio of ‘EF mean and gene mean’ and the qRT-PCR results are indicated as ‘mRNA fold-increase’ with respect to the calibrator.

### 2.3. Primer Design and Citrus Genome Support

Primers were designed using Primer 3 software [33], preferably on coding sequences on the exon-exon junction. The presence of dimers was confirmed using the Oligo Analysis Tool [34]. The genes, coded by genome position, and their related primers are listed in Table 2. The elongation factor 1 alpha (Cs8g16990) was used as a housekeeping gene to normalize the expression data.

To ensure the evaluation of genes corresponding as closely as possible to each accession, all primers were first designed on the *C. sinensis* genome [35]. Then a blast against NCBI Primer tool [36] was carried out to evaluate the conservation of the target sequence in species different from sweet orange. At the same time, the nucleotide sequence of each gene of sweet orange was searched, for homology against species deposited in the *Citrus* genome collection [37,38]. Specifically, we matched against the genomes of *C. maxima*, *C. medica*, *C. reticulata*, and *S. buxifolia*, representing the parents or the species themselves that we considered in the present study. Moreover, we also blasted against the *C. ichangensis* genome, a Papeda like *C. hystrix* and *C. latipes*. Finally, we also included the blast against *Fortunella hindsii* which is close enough to the *Citrus* species, although it belongs to another genus [39]. The CLC Sequence Viewer software [40] was used to carry out the nucleotide and aminoacidic ClustalW alignments of the genes and primers to individuate the perfect match or the eventual differences among species (Table S1 and Figure S2).

The *M. australasica* genome (SRR6188442 SRA) was downloaded in FASTQ format using the “FASTQ-dump” tool of the SRA toolkit [41] from NCBI, with the option split-files to obtain two separate files for the paired-end data. The two files were trimmed by Trim Galore [42] and aligned on the *C. maxima* reference genome [37] by Stampy [43], which is able to map reads from highly divergent species to a reference genome. The hybrid mode Bwa/Stampy [43] was used to speed up the alignment. The file obtained was sorted and indexed by SAMtools [44]. After alignment, the depth and coverage were estimated by SAMtools. To produce a FASTA format of each gene investigated, the reads mapping on each gene model were extracted with SAMtools-view and the consensus was produced with SAMtools-mpileup, BCFtools-call [45], and the vcfutils.pl concatenate process [46].

### 2.4. Image Analysis of the Petals

Photographs of the petals were taken against a green background under artificial light sources (Figure S3). As a reference for the size and color calibration, a yellow tag label was positioned close to the petals. The petals were arranged in rows, exhibiting the inner and the outer faces. Photographs were processed by Fiji distribution of ImageJ [47], and the binary images were obtained using SIOX segmentation [48]. The basic intensity quantification was done by analyzing particles as mean grey values of a 16-bit image converted to greyscale [49].

### 2.5. Statistical Analyses

Correlations among gene expressions were evaluated as mRNA-fold increases for each tissue using the Pearson method and data were considered discriminant for *p*-values ≤ 0.05.

A qualitative test was used to compare the significant differences between pigmented and non-pigmented samples for all tissues, except young leaves, stamens and styles because only one negative control was collected. The normal distribution of the expression data, based on the mRNA-fold increase values, was tested using the Shapiro-Wilk method [50]. The means of the normally distributed samples was compared by *t*-test, while the samples that were not normally distributed were tested using the Wilcoxon signed-rank test [51].

Two different Ward hierarchical clustering analyses with bootstrapped *p*-values were applied to all genes and all accessions evaluated for each tissue, except for the stamens, styles, and stigmas because of the small number of accessions (from 4 to 6). The analyses were done using the default setting by ‘pvclust’ R package [52].

Principal component analysis (PCA) was carried out on all genes and the accessions of all tissues (except for the stamens, styles, and stigmas) by the ‘prcomp’ function [53].

Partial least square regression (PLS) was carried out on all the genes of the accessions, where petals were analyzed by the ‘plsr’ function of ‘the pls’ package [54] and validated using the leaving one out ‘LOO’ method. The graphics were plotted using the ‘ggplot2’ package [55]. Social network analysis was carried out and plotted using ‘d3Network’ [56] and ‘igraph’ [57].

All statistical analyses were carried out in R ambience [58].

### 2.6. Anthocyanin and Lycopene Quantification

Anthocyanin quantification was carried out spectrophotometrically at 520 nm of absorbance (Varian UV-Vis spectrophotometer mod. Cary 100 Scan) according to the differential pH method [59]. Volumes of 2 mL of centrifuged juice (13,000 rpm for 40 min) were used for sweet oranges, while volumes of 4 mL of methanolic extract were used for the flesh of *M. australasica*. Methanolic extracts were obtained by adding 2 g of *M. australasica* flesh to 50 mL of 80% acidified methanol (0.5% HCl), stored under agitation in the dark for 3 h, and then centrifuged at 8000 rpm for 40 min. Total anthocyanins were expressed as milligrams per liter (mg/L) of cyanidin 3 glucoside.

Lycopene quantification was carried out spectrophotometrically at 503 nm of absorbance [60] by extracting carotenoid pigments [61] with 25 mL of hexane/acetone/ethanol solution mixed with 3 g of *M. australasica* flesh and, similarly, with 20 mL of hexane/acetone/ethanol solution (50:25:25) mixed with 10 mL of orange juice. Lycopene content, expressed in milligrams per liter (mg/L), was determined using the molar extinction coefficient of E^1%^_1 cm_ = 3450 [62].

## 3. Results and Discussion

### 3.1. Genome Data Support the Variability and Specificity of the Expression Analysis

The genomes of all accessions considered here are not totally or publicly available. The *M. paniculata* genome has been released but is not yet available (Unpublished data-“CREA-OFA jointly IGA-TS De novo sequencing of *M. paniculata*-ORPRAMed project”). The genome of *M. australasica* is available but it has been aligned against the *C. maxima* genome resulting in an average coverage of 38 ×, covering 269 Mb of the total 302 Mb of the reference genome.

Generally, we observed that anthocyanin biosynthesis is highly conserved in all the species we considered. This finding is supported by the high sequence homology for all structural genes.

Two considerations deserve mention: the first relates to *M. paniculata* and *S. buxifolia*, in which the primers worked correctly, even though very few nucleotidic and aminoacidic mutations were reported in the primer(s); the second relates to genes with null expressions even though no mutations were reported in the primer sequences.

*Murraya* spp. is known for its ornamental value (specifically *M. paniculata* and *M. ovatifoliolata*) and for the uses of their leaves as spices (*M. koeniji*). From a phylogenetic point of view, the relationship between the *Murraya* and *Citrus* genera is controversial, even though the *Murraya* genus is considered quite distant from *Citrus.* Recently some authors [63,64] have indicated that *M. paniculata* is more closely related to *Citrus* than previously thought. However, this suggestion has not yet been fully accepted [65]. This is the first time in which red fruits have been evaluated and genetically characterized for the presumed presence of anthocyanins. Except for PAL, which was perfectly conserved (100% homology), the other genes showed some mutations that did not affect their aminoacidic sequences (such as CHS, DFR, and ANS) and some others (such as UFGT, GST, Ruby and Noemi) showed longer mismatches that compromised not only the synthesis of anthocyanins but also their accumulation and the regulation (Table S1, Figure S2). The expression of GST was completely null (see later), and Ruby was not found in the *M. paniculata* genome. This leads us to hypothesize that the brilliant red color of the fruit may not be due to anthocyanins [30], as also reported in Table S2. Previous phytochemical studies reported that fruits of *M. paniculata* contain coumarin and coumurrayin [66], in addition to polymethoxylated flavones, flavonols, and flavanonols [67].

*Severinia buxifolia* and *S. disticha* represent unusual accessions, because *S. disticha*, in particular, accumulates anthocyanins in both mature fruits and young leaves [30] differently from the other citrus accessions. Although most of the structural genes (except ANS) showed from one to three nucleotide substitutions in the primer binding regions (but not in any case modifying the amino acid sequence), the anthocyanin pathway was fully conserved, as demonstrated by the purple pigmentations in the young leaves and in the fruit. The peculiarity of *S. buxifolia* is associated with the unusual control of anthocyanins, being due to Ruby2 [4] instead of to Ruby. Nucleotidic substitutions inducing differences in the aminoacidic sequences were found in the Noemi gene, although they did not interfere with gene expression, which was the greatest in the rind. Moreover, the nucleotide and amino acid substitutions were the same as those observed in citron, pummelo, *M. australasica*, and *C. ichangensis*, demonstrating very close conservation (Table S1 and Figure S2).

The second consideration regards the null expression of Ruby in the young purple leaves of both citrons, i.e., ‘Diamante’ and ‘Buddha’s hand’, and for *M. australasica*, despite the perfect match of the primers. We speculate that the pigmentation of the leaves of these genotypes is not under the control of Ruby. In contrast, the null expression of Ruby in the white petals of ‘Zagara bianca’ could be due to the loss of function of Ruby itself, since Ruby is transcriptionally active in lemons with purple flowers. Lastly, the missing expression of Noemi in the pigmented leaves of *C. celebica* and *M. australasica*, such as in the purple petals of ‘Incomparabile’ and ‘Red lime’, could be attributed to the fact that Noemi co-works with Ruby in the flesh [19], but probably not in the other pigmented tissues, as reported in other plants [44,45]. The missing expression of Noemi in the low-acid flesh of ‘Vaniglia sanguigno’ confirms its role as the gene responsible for the loss of acidity [19]. The primers of Noemi were specifically designed in the region that has been deleted in the low acid accessions.

### 3.2. Anthocyanin Pigmentation Is an Extremely Variable Trait among Citrus Species and Tissues

The understanding of how gene expression patterns can change among tissues and during evolution is an important question that involves both developmental and evolutionary biology [68]. Pigmentation represents an attractive model for such studies, changes in color being easy to score visually, and providing a genetic network under the control of external factors [69,70,71]. In plants, the R2R3-MYBs play a central role in regulating the spatio-temporal expression pattern of the genes involved in synthesis and accumulation of pigments, such as anthocyanins [72], condensed tannins [73], betalains [74], and copigment flavonols [75], which are the main players in determining pigmentation patterns.

In a present study, we investigated the tissue-specificity of anthocyanin pigmentation in a range of samples belonging to several species and hybrids. In modern and ancient *Citrus*, the genetic control of pigmentation is variously inherited, depending on the tissue. Pigmentation is also influenced by environmental factors, such as light [15], cold [12], and cultural practices [14,76]. To reduce the influence of such effects, which might upset inter-comparison among samples, all accessions were sampled from the same germplasm bank. Nevertheless, we cannot exclude the possibility that a part of the observed variability could be typical of our environment and so might not be evident in other citrus growing areas.

#### 3.2.1. The Pigmentation of Young Leaves Was Inherited from Citron

In the leaves of some plant species, anthocyanins are expressed throughout development (reviewed in [6]). In other species the pigments are expressed only in the early stages, disappearing later as development proceeds [77,78,79]. Meanwhile, in other species, pigmentation is specific to the later stages of leaf senescence (for woody perennials see [80], for herbaceous species see [6]). Many researchers have explored the possible functional role(s) of anthocyanins in leaves, seeking to explain the molecular mechanisms controlling this phenomenon. None of the hypotheses offered provide a unified explanation for the diverse range of environmental triggers, or for the variability in pigment location and expression in particular stages of development [81,82]. In *Citrus* and related genera, the purple pigmentation of the leaves typically characterizes the early status of development while the red coloration of large adult leaves generally decreases, eventually disappearing.

**Figure 2:**
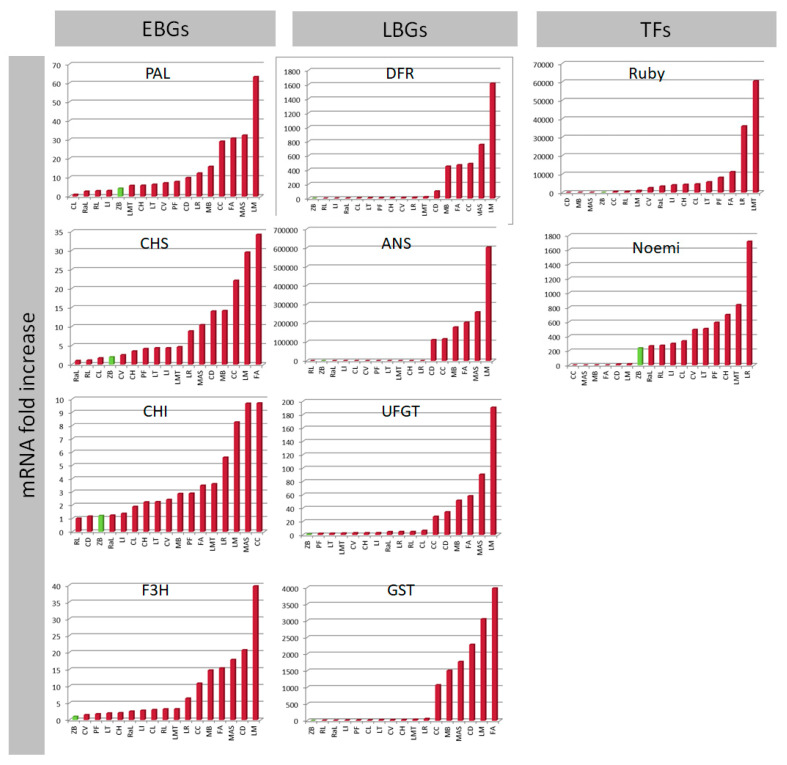
qRT-PCR expression data for all structural Early Biosynthetic Genes (EBGs) and Late Biosynthetic Genes (LBGs) and Transcription Factors (TFs) separated from young leaves. The expression levels were calculated as the mRNA fold increase. Each accession is indicated by a code: ‘Zagara bianca’—ZB, ‘Red lime’—RL, ‘Rangpur lime’—RaL, ‘Incomparabile’—LI, ‘Pink fleshed’—PF, *Citrus latipes*—CL, ‘Tahiti’—LT, *C. volkameriana*—CV, *C. hystrix*—CH, ‘Mexican lime’—LMT, ‘Lima rossa corrugata’—LR, *C. celebica*—CC, ‘Buddha’s hand’—MB, *M. australasica* var. sanguinea—MAS, ‘Diamante’—CD, ‘Meyer’—LM, ‘Femminello Adamo’—FA.

The expression data indicate that most EBGs do not usually show differences between pigmented and non-pigmented samples (Figure 2). Nevertheless, the strategic role of F3H in regulating pigmentation is confirmed, as has also been reported in bilberry [83]. Among the LBGs, UFGT and GST represent genes with higher correspondence between expression and pigmentation (Figure 2). ‘Zagara bianca’ is the negative control and, at the same time, also serves as the calibrator of expression analysis. All genes belonging to phenylpropanoid and anthocyanins biosynthesis and accumulation, help to explain the variability among accessions. From a functional point of view, UFGT represents the last gene of the biosynthesis, which induces anthocyanins to be synthesized in the cytosol, then conjugates these with glutathione and transfers them to the vacuole where the anthocyanins are accumulated. In citrus, as in other plants, the role played by of anthocyanins in the leaf vacuoles remains obscure.

**Figure 3:**
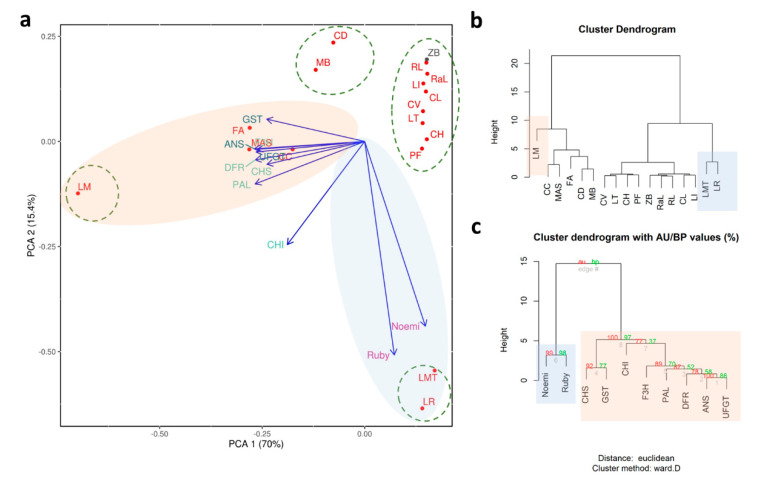
Statistical analyses carried out on accessions characterized by the pigmentation of young leaves. (**a**) Principal component analysis; cluster dendrograms based on the distribution of expression data focusing on (**b**) accessions and (**c**) genes where AU refers to Approximately Unbiased *p*-value and BP is for Bootstrap Probability value. Each accession is indicated by a code: ‘Zagara bianca’—ZB, ‘Red lime’—RL, ‘Rangpur lime’—RaL, ‘Incomparabile’—LI, ‘Pink fleshed’—PF, *Citrus latipes*—CL, ‘Tahiti’—LT, *C. volkameriana*—CV, *C._hystrix*—CH, ‘Mexican lime’—LMT, ‘Lima rossa corrugata’—LR, *C. celebica*—CC, ‘Buddha’s hand’—MB, *M. australasica* var. sanguinea—MAS, ‘Diamante’—CD, ‘Meyer’—LM, ‘Femminello Adamo’—FA.

In our study, around 85% of the variance in the pigmentation of young leaves is explained by two main components (Figure 3a). Pure citrons, i.e., ‘Buddha’s hand’ and ‘Diamante’, are separated from all the other accessions, even though they are close to citron hybrids, including the lemon group. Among these accessions ‘Femminello Adamo’ is clearly separated from the rest of the lemons because it represents the only true lemon considered in the present work, as characterized by pigmented young leaves and flowers and by an acidic juice [84]. The other lemons we considered were somatic mutations with clear phenotypic differences. Specifically, ‘Zagara bianca’ is characterized by non-pigmented leaves and petals, and ‘Pink fleshed’ which accumulates lycopene in the flesh and produces variegated fruits and leaves.

The PCA shows that ‘Meyer’ is the accession that diverges most from the others (Figure 3a,b). Presumably, this is due to the very high expression levels of some of the structural genes (such as PAL, F3H, DFR, ANS, and UFGT; Figure 2), as also emphasized in the cluster dendrogram (Figure 3c). We hypothesize that the divergence of ‘Meyer’ can be traced back to its origin, considering that it is the only accession that has *C. maxima* as a parent [84]. There are a number of nucleotide substitutions along the sequences of the genes we considered, though these do not lie within the primer sequences.

The regulatory genes Ruby and Noemi induce a divergence between ‘Mexican lime’ and ‘Lima rossa corrugata’, on the one hand, and ‘Buddha’s hand’ and ‘Diamante’ on the other (Figure 3a,b). The expression levels of first two accessions are very high (a 60,000-fold increase for Ruby and a 1700-fold increase for Noemi) (Figure 2). Previous studies have suggested that ‘Mexican lime’ is a direct hybrid of citron and *C. micrantha* [84,85,86]. This is the only accession with both parents being characterized as having purple leaves, likely explaining the extreme separation from other accessions. Moreover, Ruby is correlated only with Noemi and Noemi is negatively correlated with GST (Table S3), leading us to hypothesize that the accumulation of anthocyanins in the young leaves is not under the direct control of Ruby and Noemi. This interpretation is also supported by the expression in ‘Zagara bianca’ and by the lack of expression in citrons and *M. australasica* (Figure 2). Ruby is known to be cold-dependent [12]. In contrast, the leaf pigmentation in *Citrus* requires further investigation, with the available evidence suggesting this trait is independent of temperature. The young leaves of all the accessions we evaluated are typically purple during the vegetative season, which is in springtime. As also reported in peach, we cannot exclude the possibility that a different MYB-like could be responsible for regulating anthocyanin accumulation in vegetative organs, such as leaves [82]. Phenotypic observations show that, among the four true *Citrus* (pummelo, mandarin, citron, and *C. micrantha*), only those species with citron and *C. micrantha* as parents are characterized by purple young leaves, not considering their related genera. We suggest that species-specific transcription factors could be responsible for the inheritance of the trait for purple pigmentation in the young leaves. Such is demonstrated for the strategic role of Ruby2 exclusively in the leaves of *S. buxifolia* but not in its (purple) fruit [4]. In Rosaceae it has been clearly demonstrated that MYB I is mainly responsible for anthocyanin accumulation in the fruits, while MYB II regulates anthocyanin accumulation in the leaves [82].

#### 3.2.2. The Molecular Mechanism Controlling the Pigmentation of Petals in *Citrus* Is Deeply Articulated and Unusual

In *Citrus*, the anthocyanin pigmentations of young leaves and of petals generally characterize the same species. Indeed, it is unusual to find accessions with purple young leaves and white flowers, or vice versa. Despite this, our expression analyses in association with image analyses, indicate that the pigmentation of petals is under the control of a complex genetic machine.

**Figure 4:**
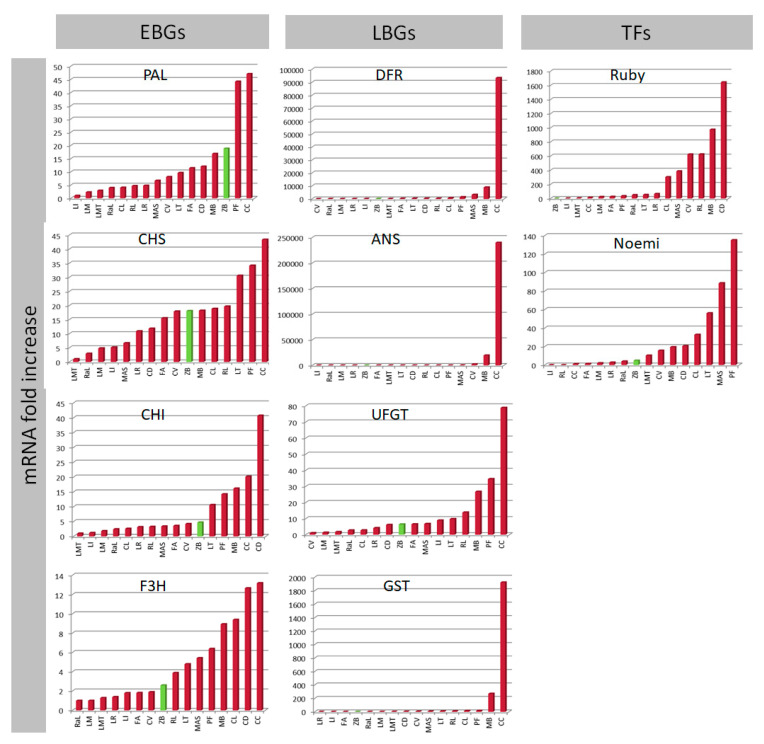
The qRT-PCR expression data of all structural genes: Early Biosynthetic Genes (EBGs), Late Biosynthetic Genes (LBGs) and Transcription Factors (TFs) analyzed in petals. The expression levels are calculated as the mRNA fold increase. Each accession is indicated by code: ‘Lima rossa corrugata’—LR, ‘Incomparabile’—LI, ‘Femminello Adamo’—FA, ‘Zagara bianca’—ZB, ‘Rangpur lime’—RaL, ‘Meyer’—LM, ‘Mexican lime’—LMT, ‘Diamante’—CD, *C. volkameriana*—CV, *M. australasica* var. sanguinea—MAS, ‘Tahiti’—LT, ‘Red lime’—RL, *Citrus latipes*—CL, ‘Pink fleshed’—PF, ‘Buddha’s hand’—MB, *C. celebica*—CC.

By browsing our expression results for petals, no differences are observed between pigmented accessions and ‘Zagara bianca’ (Figure 4). Even though the purple petals of ‘*C. celebica*’ represent the sample with the highest expression of PAL. ‘Zagara bianca’, whose petals are completely white, show an expression level 47-fold higher than of the purple petalled ‘Incomparabile’. Similarly, the EBGs are not much different between the pigmented and non-pigmented samples. In fact, for CHS, CHI and F3H, although more highly expressed in *C. celebica* than in ‘Zagara Bianca’ (about a 45-fold difference), F3H is still highly expressed in comparison with several of the pigmented accessions. Surprisingly, the LBG expressions are not very different between pigmented and non-pigmented samples.

**Figure 5:**
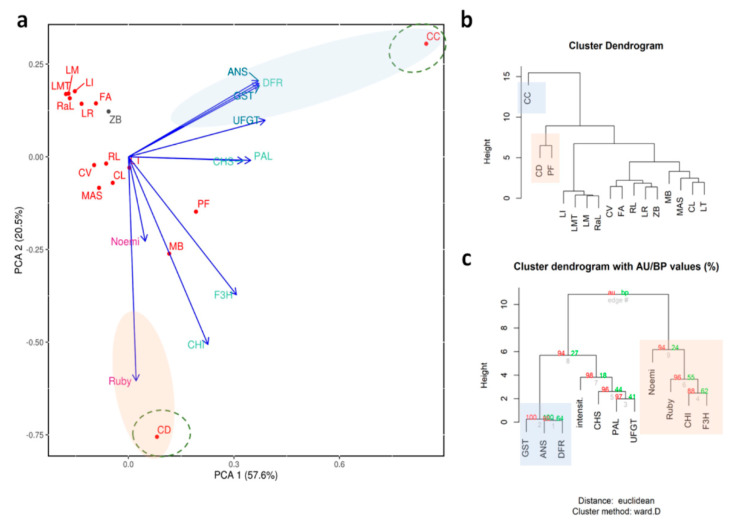
Statistical analyses carried out on accessions selected for their petal pigmentation. (**a**) Principal components analysis; cluster dendrograms based on the distributions of expressions focused on (**b**) accessions and on (**c**) genes where AU refers to Approximately Unbiased *p*-value and BP is for Bootstrap Probability value. Each accession is indicated by code: ‘Lima rossa corrugata’—LR, ‘Incomparabile’—LI, ‘Femminello Adamo’—FA, ‘Zagara bianca’—ZB, ‘Rangpur lime’—RaL, ‘Meyer’—LM, ‘Mexican lime’—LMT, ‘Diamante’—CD, *C. volkameriana*—CV, *M. australasica* var. sanguinea—MAS, ‘Tahiti’—LT, ‘Red lime’—RL, *Citrus latipes*—CL, ‘Pink fleshed’—PF, ‘Buddha’s hand’—MB, *C. celebica*—CC.

**Figure 6:**
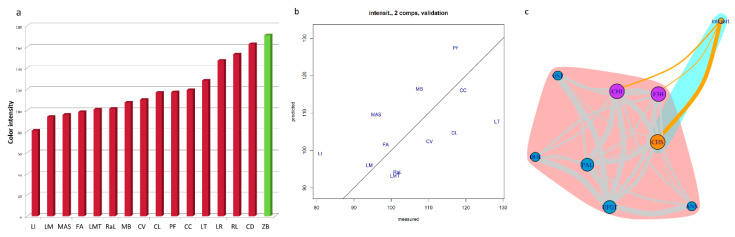
Statistical analyses of the quantitative trait of anthocyanins accumulation in petals. The accessions are indicated with codes: ‘Incomparabile’—LI, ‘Meyer’—LM, *M. australasica* var. sanguineaMAS, ‘Femminello Adamo’—FA, ‘Mexican lime’—LMT, ‘Rangpur lime’—RaL, ‘Buddha’s hand’—MB, *C. volkameriana*—CV, *Citrus latipes*—CL, ‘Pink fleshed’—PF, *C. celebica*—CC, ‘Tahiti’—LT, ‘Lima rossa corrugata’—LR, ‘Red lime’—RL, ‘Diamante’—CD, ‘Zagara bianca’—ZB. (**a**) Bar plot of petal color intensity measured by image analysis on petals as the intensity in grayscale (whitest has the highest value) reported for each accession. (**b**) Validation plot of the partial least square regression (PLS) model for petal pigmentations. Values measured by image analysis of petals versus predicted values from gene expression using the first two components of the model. (**c**) The network of gene expressions in petals involved in pigmentation measured as the color intensity. The thickness of edges represents the Pearson’s Correlation Coefficient value, the node dimension represents the weight of the node in the network, the color of the edge represents the relationship among genes in grey and with the phenotypical trait in dark orange. The node colors represent the subgroups and the halo represents community detection, based on edge-betweenness.

About 78% of the total variance among samples is attributed to the two main components (Figure 5a), also showing a large divergence for *C. celebica* (Figure 5b). This could be due to the higher expressions of DFR, ANS, and GST compared to the other accessions (the fold increases are 240,000, 93,000 and 1900 mRNA, respectively) (Figure 4) supported by their high correlations (Table S3) and to divergence with respect to the other genes (Figure 5c). The exclusivity of *C. celebica* can be ascribed to its origins: *C. celebica* derives from *C. micrantha* (similar to ‘Mexican lime’), even though it is sited in the opposite direction (Figure 5a), confirming a previous report [86]. Grayscale image analysis of the petals reveals the highest value of color intensity is 171, for ‘Zagara bianca’, the whitest among the accessions. The darkest petals are those of ‘Incomparabile’, recording a relative color intensity value of 81 (Figure 6a). The other accession values decreased gradually between these maximum and the minimum values of the range.

Surprisingly, petal color intensity does not show a significant direct relationship with the expression data. To better investigate this, two different approaches were used, namely PLS (Figure 6b) and social network analysis (Figure 6c). During the development of the PLS model, four accessions appeared as outliers—the negative control ‘Zagara bianca’ and three of the pigmented samples, ‘Diamante’, ‘Red lime’, and ‘Lima rossa corrugata’. The exclusion of these accessions from the dataset allowed the construction of a simple model for predicting color intensity. This is represented by the PLS validation plot (Figure 6b). The graph shows the comparison between the intensity calculated through image analysis (abscissa) vs. the intensity predicted by the PLS analysis (ordinate). The input data for this are the expression values just for the first two components, which explain 57% of the intensity variance (Table S4). We cannot exclude the possibility that the unexplained variance may be due to the thickness of the petals of all the species. These covered a wide range of thickness values and were also different in size, which could also reduce the sensitivity of the analysis. Using the subset defined in the PLS, a social network analysis was set up (Figure 6c), which emphasizes the relationship between petal intensity and the expressions of the CHI, CHS and F3H genes. These results are consistent with the weights that the expressions of these genes had in the PLS model (Table S4). In the paired *t*-test (Table S5), CHI, CHS, and F3H did not show differences between the pigmented and non-pigmented samples. The social network analysis indicates that the fine regulation of anthocyanins accumulation in petals seems to be modulated by these three genes.

Focusing on the transcription factors, Ruby and Noemi showed significant differences in expression between pigmented and non-pigmented petals (Table S5). Nevertheless, as evidenced in the statistical analyses (Pearson’s correlation, Table S3; PLS and network analyses—Figure 6b,c), Ruby alone cannot be considered the transcription factor controlling the pigmentation in petals. For example, in petunia flowers, the pigmentation of petals is under the control of a complex machinery, including also ATPases required for vacuolar hyperacidification linked with the pH of petals [87,88,89,90], in addition to R2R3-MYB, that together govern anthocyanin synthesis under a range of spatio-temporal circumstances [91,92]. For Noemi, the importance of ion exchange and the role of vacuolar ATPase in petals are well known [93]. Therefore, we cannot exclude the possibility that a tissue-specific ATPase works together with Noemi and Ruby to regulate the variability in anthocyanin pigmentation. This possibility is currently being evaluated.

#### 3.2.3. The Pigmentation of the Rind Is Independent of the Genetic Basis of the Parents and Is under the Control Mainly of External Factors and Fruit Developmental Stage

The pigmentation of the fruits of *Citrus* and related genera is very variable, considering that either or neither or both the rind and the flesh can be pigmented. Additionally, many factors can influence the pigmentation including low temperature, exposure to light and stage of development [10,14].

The samples in our study embrace a wide range of variation in fruit pigmentation across a number of species, including of sweet oranges, mandarin types, several hybrids of citron, also including other genera such as *Microcitrus*, *Murraya*, and *Severinia* (Table 1).

**Figure 7:**
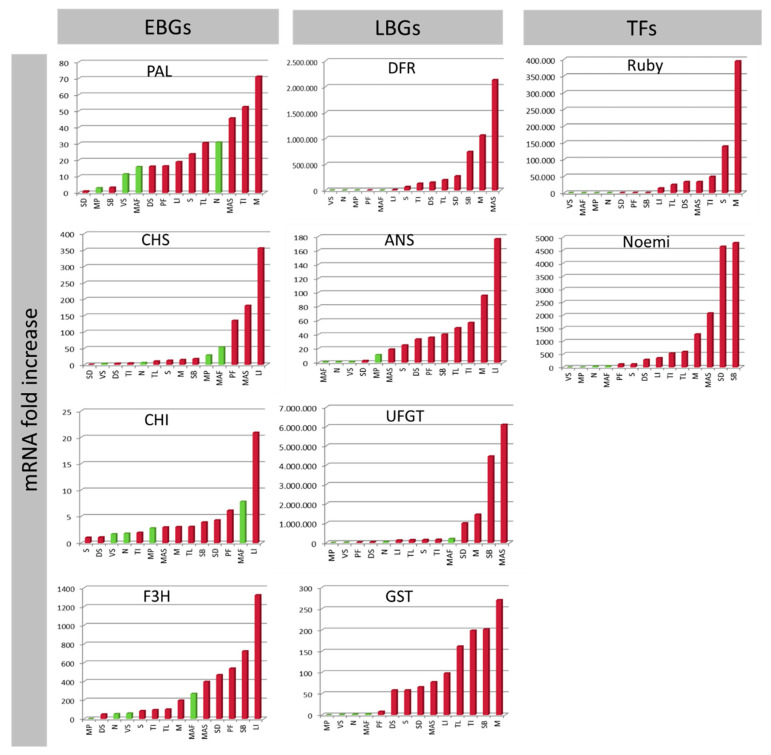
qRT-PCR expression data for all structural genes in the rind separated into Early Biosynthetic Genes (EBGs), Late Biosynthetic Genes (LBGs) and Transcription Factors (TFs). The expression level was calculated as the mRNA -fold increase. Each accession is coded: *M. paniculata*—MP, ‘Vaniglia sanguigno’—VS, ‘Navel’—N, ‘Faustrime’—MAF, ‘Pink fleshed’—PF, ‘Doppio sanguigno’—DS, ‘Sunred’—S, *S. disticha*—SD, *M. australasica* var. sanguinea—MAS, ‘Incomparabile’—LI, ‘Tarocco Lempso’—TL, ‘Tarocco Ippolito’—TI, *S. buxifolia*—SB, ‘Moro’—M.

The expression data show that, as with other tissues, PAL is not expressed differentially among samples (Figure 7). Indeed, ‘Navel’ showed an expression level comparable to ‘Tarocco Lempso’ (about a 31-fold increase) which is deeply pigmented [14] and the expression in ‘Faustrime’ is similar to that in ‘Doppio sanguigno’—the first not at all pigmented but the second is very much pigmented. Among EBGs, the CHS in *M. paniculata* and ‘Faustrime’ is highly expressed compared to in *S. disticha*, *S. buxifolia*, ‘Sunred’, and ‘Moro’, which are the accessions showing high pigmentation in the rind. Similarly, F3H did not reveal any interesting differences between the pigmented and non-pigmented samples. A completely different trend and expression levels characterize the LBGs, strategically involved in the control of the pigmentation of the rind. For example, the expression of DFR in *M. australasica*, with highly pigmented rind, is the highest with a 2.5 million-fold increased expression level. Similarly, the expression of UFGT, the last enzyme of the phenylpropanoid biosynthesis, showed around a 6 million-fold increase in expression in *M. australasica* compared to *M. paniculata*, the accession with the lowest expression of UFGT in the rind. Additionally, very interesting are the expressions of GST, Ruby and Noemi, these being highly and exclusively expressed in the pigmented samples, compared with the negative controls. Generally, we can assume that the rind pigmentation is under the control of DFR, ANS, GST, Ruby and Noemi, as also shown in the *t*-test analysis (Table S5). These presumably represent the major genes determining rind pigmentation. The expression patterns of Ruby and Noemi mirrored the differences between the pigmented and non-pigmented samples. In terms of the values of the mRNA fold increase, they were higher for Ruby than for Noemi.

**Figure 8:**
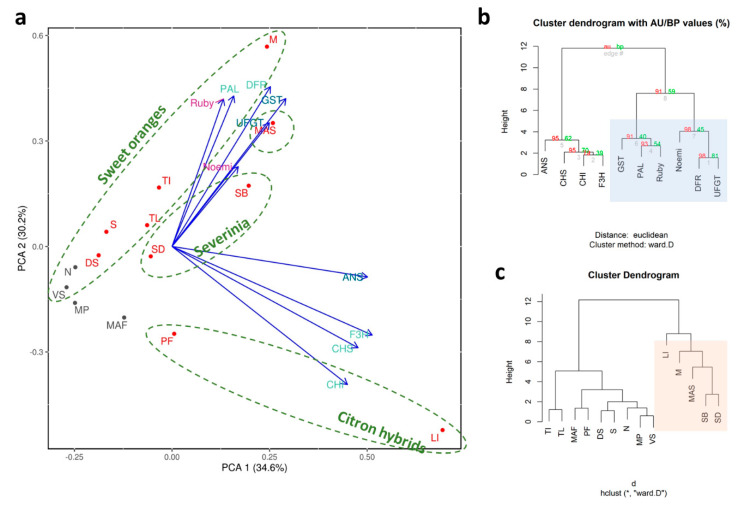
Statistical analyses carried out on accessions selected for their rind pigmentation. (**a**) Principal component analysis, cluster dendrogram based on the distribution of expression data focused (**b**) on genes where AU refers to Approximately Unbiased *p*-value and BP is for Bootstrap Probability value; and (**c**) on accessions. Accessions are coded: *M paniculata*—MP, ‘Vaniglia sanguigno’—VS, ‘Navel’—N, ‘Faustrime’—MAF, ‘Pink fleshed’—PF, ‘Doppio sanguigno’—DS, ‘Sunred’—S, *S disticha*—SD, *M. australasica* var. sanguinea—MAS, ‘Incomparabile’—LI, ‘Lempso’—TL, ‘Ippolito’—TI, *S. buxifolia*—SB, ‘Moro’—M.

The separation between pigmented and non-pigmented rinds is clearly seen in the PCA, where all negative controls are near the origin of the plot (Figure 8a). Around 65% of the variability is explained by the two main components, these are almost equal with 34.6% and 30.2% of total variability. The PCA (Figure 8a) and the cluster dendrogram (Figure 8b) show how genes with higher expressions, such as UFGT (a 6 million-fold increase), followed by DFR (a 2 million-fold increase), and Ruby (a 390,000-fold increase) characterize the samples with deep external color, such as *M. australasica*, ‘Moro’, and *S. buxifolia*. The PCA (Figure 8a) and the cluster dendrogram (Figure 8c) also support the interspecies discrimination, confirming that the external pigmentation of fruits of sweet orange, citron, and the citron hybrids, *S. disticha* spp. and *M. australasica*, is very variable. The rind of blood oranges becomes purple during maturation, likely under the control of low temperatures. The fruits of *S. disticha* spp. are externally pigmented at maturity but this is independent of temperature and light [4]. The fruits of *M. australasica* var. sanguinea are pigmented; they are also very small and maintain their red color throughout development. Finally, accessions with citron as a parent (such as ‘Incomparabile’) are characterized by having pigmented fruits only when they are very small, the pigmentation then decreases, disappearing altogether as the fruit enlarges. We hypothesize that the separation of ‘Incomparabile’ and ‘Pink fleshed’ from all the other accessions can be ascribed to stage of development and maturity, very precocious compared to the mature fruits of all of the other accessions.

#### 3.2.4. Flesh Pigmentation Is Exclusive to Purple Sweet Oranges and to *M. australasica* and Is Differently Correlated with the Acidity Trait

Flesh pigmentation is a very unusual trait among *Citrus* species, being exclusive to a subset of sweet orange varieties including Moro, Tarocco, and Sanguinello and of hybrids with one of these as a parent. Even though pummelo represents one of the parents of the sweet orange, where a very ancient and original variety has been reported to be externally pigmented, the pigmentation of the flesh is specific only to sweet orange. The red color of the flesh of *M. australasica* var. sanguinea represents another example of inner fruit pigmentation, but different from sweet orange. In fact, the fruits of *M. australasica* are characterized by a homogeneous distribution of pigmentation in the flesh, independent of ripening. In sweet orange the pigmentation of the flesh increases during the fruit ripening. This is the first time that a genetic overview has been conducted on a subset of accessions, that include sweet orange varieties with different levels of pigmentation, and other *Citrus* species.

**Figure 9:**
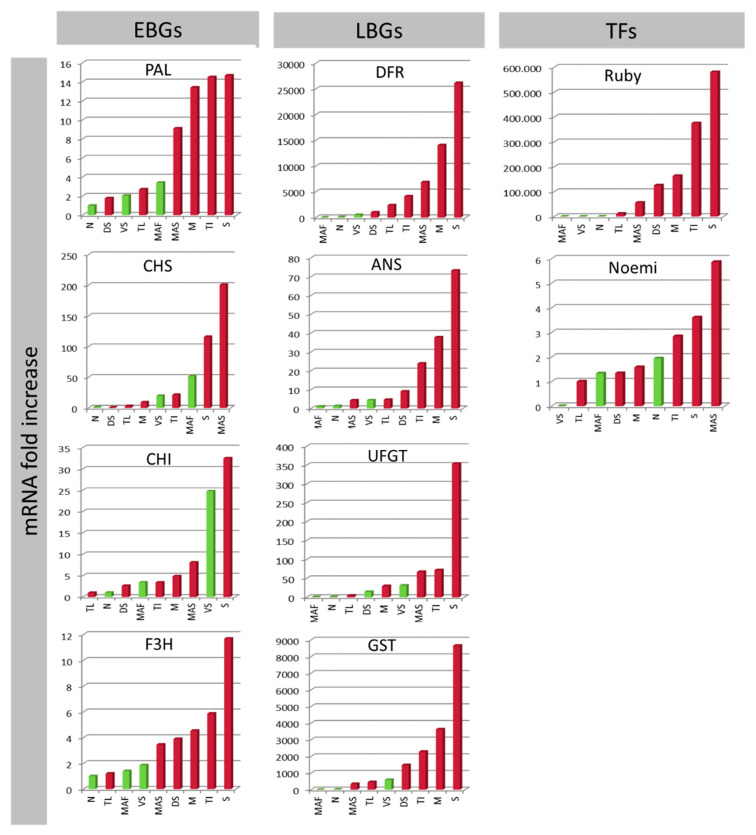
qRT-PCR expression data for all structural genes separated into Early Biosynthetic Genes (EBGs), Late Biosynthetic Genes (LBGs), and Transcription Factors (TFs) in the flesh of *M. australasica* and *Faustrime*, and of the juices of all other accessions. The expression level was calculated as the mRNA-fold increase. Accessions are coded: ‘Faustrime’—MAF, ‘Navel’—N, *M. australasica* var. sanguinea—MAS, ‘Tarocco Lempso’—TL, ‘Vaniglia sanguigno’—VS, ‘Doppio sanguigno’—DS, ‘Tarocco Ippolito’—TI, ‘Moro’—M, ‘Sunred’—S.

Expression analysis of the genes involved in the early steps of biosynthesis shows no particular difference between the pigmented and non-pigmented samples (Figure 9). The expression of PAL in ‘Navel’, ‘Vaniglia sanguigno’, and ‘Faustrime’ is very similar to that in ‘Doppio sanguigno’ and ‘Tarocco Lempso’ blood oranges, which are typically pigmented. Similarly, the expression of CHS in ‘Faustrime’ is higher than in ‘Tarocco Ippolito’. Moreover, the expression of CHI in the flesh of ‘Vaniglia sanguigno’ is higher than those in the pigmented accessions. The expression of F3H in ‘Tarocco Lempso’ is comparable to that of the negative controls.

In contrast to this is the LBG trend. DFR was the highest expressing of the LBG genes with an expression showing about a 26,000-fold increase, followed by GST with 8600-fold, both in ‘Sunred’ (Figure 9). The DFR showed very high expressions (1000- to 25,000-fold increases) in all the pigmented samples, compared with the negative controls. In particular, ‘Sunred’ juice showed around 25,000-fold increased expression, compared with the negative controls ‘Faustrime’, ‘Navel’, and ‘Vaniglia sanguigno’. Our results support the strategic role of DFR in the anthocyanin pathway, as previously reported [12,25,94]. For ANS and UFGT we observed some irregularities, mainly for ‘Vaniglia sanguigno’. Our results show an expression of ANS in ‘Vaniglia sanguigno’ comparable to that in *M. australasica* and ‘Tarocco Lempso’ (both highly pigmented), even though the expression level of ANS in ‘Vaniglia sanguigno’ is very low. The expression levels of UFGT in ‘Vaniglia sanguigno’ and ‘Moro’ juice are very similar (about 30-fold increases). Lastly, the expression of GST in ‘Vaniglia sanguigno’ is also very similar to that in ‘Tarocco Lempso’ and *M. australasica*. Anthocyanin and lycopene quantifications in ‘Vaniglia sanguigno’ show that the red pigmentation could be due to lycopene (Table S2) and not to anthocyanin, as previously proposed [14,27]. We cannot exclude the possibility that the expression, mostly of LBG and GST, is because the primers were designed in a shared region, although these genes could be truncated somewhere along the sequence.

The role of GST is strategic. It is a very large family of about 10 classes, these differing both structurally and functionally. This family is implicated in the vacuolar sequestration of flavonoids, as well as in xenobiotic detoxification (reviewed in [95]). Less recently, it has been noted how GST tissue-specific expression can be due to exposure to chemical treatments and to biotic or abiotic stresses [6]. We selected the member belonging to the phi class, which is involved in anthocyanin accumulation in blood orange flesh [96,97].

From a statistical point of view, the qualitative difference between the pigmented and non-pigmented accessions is due to PAL and Ruby (Table S5), the major genes for flesh tissue. The crucial role of PAL was firstly explained by Lo Piero et al. [10], who showed how PAL expression increased in Tarocco under exposure to low-temperatures. This suggests its role as a cold protector [98]. Later on, after the construction of a suppression library, the overexpression of PAL in Moro was further demonstrated to be one of the genes differentially expressed [25].

**Figure 10:**
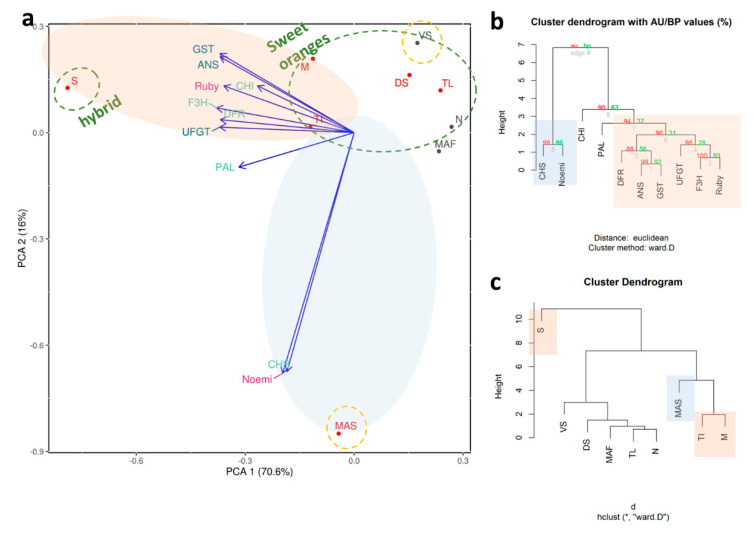
Statistical analysis of accessions selected for their pigmentation in flesh and juice. (**a**) Principal component analysis, cluster dendrogram based on the distribution of expression data focused (**b**) on genes and (**c**) on accessions. Accessions are coded: ‘Faustrime’—MAF, ‘Navel’—N, *M. australasica* var. sanguinea—MAS, ‘Tarocco Lempso’—TL, ‘Vaniglia sanguigno’—VS, ‘Doppio sanguigno’—DS, ‘Tarocco Ippolito’—TI, ‘Moro’—M, ‘Sunred’—S.

Overall, more than 86% of the variance among the samples is explained by the first two principal components (Figure 10a). In particular, the deep and intense purple pigmentation of ‘Moro’, ‘Tarocco Ippolito’ and ‘Sunred’ is due mainly to F3H, to all LBGs, to GST and to Ruby (Figure 10b,c). According to previous reports, the anthocyanin content varies between approximately 6 and 25 mg/L for the ’Sanguigno’, ‘Sanguinello‘, and ‘Tarocco’ accessions, from 50 to 120 mg/L for Moro, and from 450 to 680 mg/L for mandarin-like hybrids [14,28]. The expression of Ruby is the highest among the genes we consider in this study (Figure 9). From a 580,000- to a 11,000-fold-increase, the expression of Ruby is shared among all of the pigmented samples. The peculiarity of the Ruby gene is described in [12], which reports how a Copia-like retrotransposon, inserted upstream in the Ruby gene, induces its expression, controlled mostly by cold stress. The cold-induced synthesis and accumulation of anthocyanins has been reported previously [14].

From a genetic point of view, the PCA (Figure 10a) and the cluster dendrogram (Figure 10c) show that ‘Sunred’ (deeply pigmented) is far from the sweet oranges group. Among them, ‘Moro’ and ‘Tarocco Ippolito’ (highly pigmented) are located in a central position with respect to ‘Doppio sanguigno’ and ‘Tarocco Lempso’ (less pigmented). We suggest this could be due to the hybrid nature of ‘Sunred’, considering that it is derived from a cross between ‘Oroval’ clementine and ‘Moro’ orange. *M. australasica* var sanguinea is clearly far from the other samples, because it belongs to a different genus. It is interesting to observe that the collocation of ‘Vaniglia sanguigno’ and ‘*M. australasica*’, on opposite extremes of the second dimension, is presumably due to the strategic role played by Noemi, the bHLH transcription factor that, together with Ruby, controls anthocyanin pigmentation [19]. The peculiarity of Noemi is also due to the fact that it mainly represents the gene that, if specifically interrupted, causes a loss of function of the acidic trait, making *Citrus* fruits low acid [19]. The primers used for expression analysis were designed in the interrupted region, to investigate both traits simultaneously, the pigmentation and the acidity. In fact, ‘Vaniglia sanguigno’ is a sweet (low acid) variety, with a pH of 6 and a titratable acidity of 0.2 [99]. The qRT-PCR expression data show a completely null expression for Noemi (Figure 9). Conversely, the expression of Noemi is reported for all other accessions considered in the study (Figure 9), even though not necessarily linked with pigmentation (see, for example ‘Faustrime’ and ‘Navel’ whose expressions are similar to blood oranges). Otherwise, *M. australasica* represents the most acidic sample among those considered, with its citric acid content higher than that of the other *Citrus* fruits [100]. *Microcitrus* is commonly known as ‘lemon cavial’ due to the spherical shape of vesicle juice and to the high acidity typical of lemon. The interconnection between anthocyanin pigmentation and the low acid trait has been reported [19], demonstrating how, in lemon and in other species derived from citron, the purple pigmentation in the leaves and petals is strictly correlated with the acidity of the fruit—an exception is sweet orange, whose pigmentation is fruit-exclusive. Therefore, its provisional role (pigmentation of vegetative tissues means acidic fruit) cannot yet be concluded, not even for a particular Tarocco clone, ‘Ferreri’, which is anthocyanin-rich in the flesh and also low acid [14,19].

#### 3.2.5. The Pigmentation of the Stamens and Styles Is Species-Specific, while the Red Color of the Stigmas is Genotype Dependent

Pigmentation represents a suitable model for studying how new patterns are generated along the evolution of species, as previously demonstrated in *Drosophila* wings whose pigmentation contributes to the separation of species [70], or in mammals where the diversification of coat color results in a strategic study of adaptation mechanisms [71]. In plants, the pigmentation is generally related to reproduction [101] and to adaptation to different growth conditions [102]. The development and regulation of flower color are influenced by many internal and external factors. Generally, flower tissues have variable pigmentation according to season, which depends also on environmental conditions and on biotic and abiotic stresses [102]. Therefore, understanding the mechanism of color development and its regulation provides an important theoretical platform, for the cultivation and improvement of new colors in various plant tissues, not having an exclusive ornamental value [103]. Moreover, the use of flower tissues as potential antioxidant sources has been studied in depth in saffron, where the stigmas, tepals, styles, and stamens represent antioxidant sources that may be used as functional ingredients [104].

Even though red flowers have evolved repeatedly in plants, as well as in *Citrus*, the color of the flowers represents an impressive trait, mostly for accessions of interest for their ornamental value. However, little is known about the pigmentation of stamens, styles, and stigmas, either from a phenotypic or from a genetic point of view. To the best our knowledge, this is the first time the different tissues of flowers have been characterized in *Citrus* from a genetic point of view, evaluating the role of anthocyanin’s biosynthetic and regulatory genes. *Citrus* species generally have perfect flowers comprising an androecium (the male reproductive part), made up of 20–40 stamens topped by white or yellow anthers containing the pollen. Meanwhile, the gynoecium (the female reproductive part) comprises a pistil including (from bottom to top) an ovary, a style, and a stigma.

**Figure 11:**
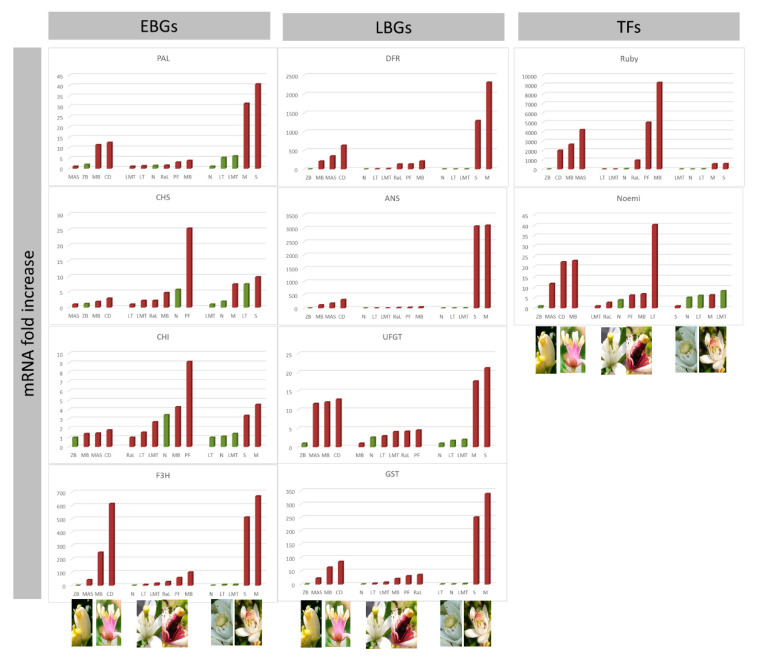
qRT-PCR expression data for all structural genes separated into Early Biosynthetic Genes (EBGs) and Late Biosynthetic Genes (LBGs) and Transcription Factors (TFs) in stamen, style, and stigma. The expression levels were calculated as mRNA-fold increases. Each accession is indicated by a code: ‘Zagara bianca’—ZB, *M. australasica* var. sanguinea—MAS, ‘Buddha’s hand’—MB, ‘Diamante’—CD, ‘Navel’—N, ‘Tahiti’—LT, ‘Mexican lime’—LMT, ‘Pink fleshed’—PF, ‘Rangpur lime’—RaL, ‘Moro’—M, ‘Sunred’—S.

The number of accessions considered to carry out a transcriptional analysis of flower tissues limits the opportunity for cluster and PCA analyses, as carried out for the other tissues of our study. Hence, this must be considered only a pilot study. Nevertheless, firstly, for the stamens and styles no differences were observed between the negative controls and the pigmented samples for PAL and EBGs, except for F3H (Figure 11). For example, ‘Pink fleshed’ is the accession with the highest expression of CHS and CHI in the style, but the negative control ‘Navel’ has an expression level higher than the other pigmented accessions. Similarly, in the stamen, CHS did not show any particular differences among samples. In fact, the stamen of ‘Zagara Bianca’ showed the same expression level as *M. australasica* (a one-fold increase) - the former non-pigmented and the latter highly colored. As for the other tissues, LBGs (except UFGT for the style) showed preferential high expressions for all pigmented samples compared to negligible expressions in the negative controls, ‘Zagara bianca’ and ‘Navel’, independent of the expression level (Figure 11). Especially for the stamens, the large difference in expression between the purple samples and ‘Navel’, supports the hypothesis that all LBGs are strictly interdependent in biosynthesis and translocation of anthocyanins. Among the transcription factors, only the stamens are under the control of Ruby and Noemi, even though the expression of the latter is lower (Figure 11). The stamens of the citrons ‘Diamante’ and ‘Buddha’s hand’ and of *M. australasica* are highly expressed compared to a very negligible expression in ‘Navel’ (from a 2000- to a 4000-fold increase for Ruby, from a 10- to a 20-fold increase for Noemi). These results suggest a putative relationship between Ruby and Noemi, as observed exclusively for the rind (Figure 4), even though the functional role of Noemi in these tissues remains uncertain. Some of these evaluations are in progress. The roles of Ruby and Noemi in the styles are less clear, considering for example the higher expression in ‘Navel’ compared to ‘Tahiti’ and ‘Mexican lime’, for Ruby, and ‘Mexican lime’ and ‘Rangpur lime’ for Noemi (Figure 11).

The stigmas represent an interesting exception, because all EBGs (except CHS) and LBGs are highly expressed in ‘Moro’ and ‘Sunred’ compared to the negative controls ‘Navel’, ‘Mexican lime’, and ‘Tahiti’ (Figure 11). The stigmas represent the only tissues in which the entire pathway from phenylpropanoid to anthocyanin biosynthesis and accumulation work together to explain the genotypically interconnected genetic control. In the framework of regulatory genes, only Ruby explains the differences between the pigmented and non-pigmented samples (Table S5), even though with a lower expression level compared to the styles and stamens (Figure 11). Moreover, from a statistical point of view, ANS and Ruby represent the two major genes responsible for the variability between pigmented and non-pigmented samples (Table S5), as also supported by the Pearson coefficients, which are highly significant (≥0.88) for all genes except for CHS (Table S3).

Among *Citrus* the red pigmentation of the stamens, styles, and stigmas is very interesting. The first phenotypic observation is that there is no correspondence or relationship with one another. The pigmentation of stamens is almost exclusively in citron and *M. australasica* and we found this to be very stable and independent of environmental conditions. The purple pigmentation of the styles is most frequent among citron and lemons but it is highly dependent on light. Interestingly, flowers, with abnormal development characterized by a protrusion of the style, show an unconventional blood pigmentation (Figure S4). Generally, we suppose that the pigmentation of the stamens and styles (except for *M. australasica*) has been inherited by citron, even though not stably, as observed for young leaves and petals. Not all accessions with pigmented leaves and petals show colored styles and stamens.

In contrast, the pigmentation of the stigmas is an exception and is genotype-dependent. The pigmentation of the stigmas is unique and exclusive to ‘Moro’ and ‘Sunred’. The stigmas of the other pigmented and non-pigmented sweet orange varieties showed no phenotypic pigmentation (Figure S5). The pigmentation of stigmas is very specific and represents a new phenotypic marker. The observation of the stigmas, in addition to the investigation on a genotype-specific single nucleotide polymorphism [105], helps clarify the origins of ‘Sunred’ (initially labeled as a ‘Tarocco’) as a ‘Moro’ hybrid [29].

## 4. Conclusions

**Figure 12:**
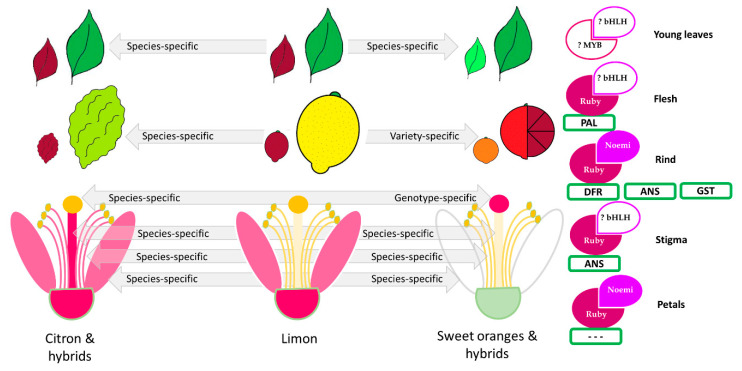
A model illustrating the putative inheritance of anthocyanin pigmentation in tissues of the main *Citrus* species and hybrids considered in this study. A scheme of leaves, fruits, and flowers tissue pigmentation is shown. A representation of transcription factors (Ruby and Noemi), biosynthetic genes (PAL, dihydroflavonol 4-reductase (DFR), anthocyanidin synthase (ANS), glutathione S-transferase (GST)) and their relationship based on the qualitative statistical analysis describing the difference between pigmented and non-pigmented samples in tissues is presented.

For the first time in *Citrus* and related genera, a wide selection of pigmented tissues has been characterized genetically and phenotypically. A hypothesis is proposed regarding the genetic inheritance of anthocyanin pigmentation in leaves, petals, flesh, rind, stamens, styles, and stigmas (Figure 12). We find that:

1. The pigmentation in young leaves and petals depends on citron. In species different from *Citrus*, the purple color in both tissues is not always correlated, such as in *Severinia* (purple young leaves and white flowers). Ruby represents the MYB transcription factor that controls the pigmentation of petals, but not of leaves.
2. The pigmentation of fruit tissues has been gained and lost frequently through the history of *Citrus*, but stably characterizes blood oranges. The control of the variability in the flesh and rind represents one of the main traits sought by consumers and producers, and one of the focuses of breeding programs all over the world.
3. The pigmentation of stamens and styles has given rise to the citron parent, but this trait is genetically less stable than in leaves and petals. Ruby and Noemi also control the pigmentation of stamens, in species different from citron and its hybrids, such as in *Microcitrus* for stamens.
4. The pigmentation of the stigma is Moro-dependent, the only genotype-dependent trait, representing a new strategic phenotypic marker. The study of light and cold-dependent control of stigma pigmentation is similar to that known in fruits, and is currently under evaluation.

A more thorough investigation of the promoter regions, as well as of transcription factors other than from Ruby and Noemi, is need if we are to better explain which mutations and molecular mechanisms dominate in the control of pigmentation in all tissues. Fuller understanding of the *Citrus* genome will help identify the specific genes forming the WMBW complex that, under various rearrangements, may provide a punctilious genetic basis to explain the generation of new patterns of pigmentation in all species.

## Figures and Tables

**Table 1:** Tissues and species evaluated in the study. The origins of the species and the hybrid for each accession are reported, as well as the accession code used in the qRT-PCR data, the references and the main traits characterizing each accession. Bright green boxes are used for negative controls, bright red boxes are used for anthocyanin-rich tissues. The corresponding light green and light red boxes refer to unpigmented and pigmented tissues collected for the expression analysis. Green boxes with asterisks refer to lycopene-rich tissues. ^#^ The pigmentation of *S. disticha* fruits could be due to the seedling origin of the accession located in the ‘Council for Agricultural Research and Economics’ bank germplasm.

							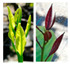	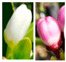	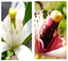	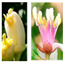	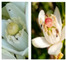	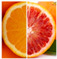	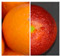
	Taxonomic Classification	Species & Hybrid	Accession	Accession Code	Reference	Main Traits	Tissues						
							Young leaf	Petal	Style	Stamen	Stigma	Flesh/Juice	Rind
**Sweet oranges & hybrids**	*C. reticulata x (C. reticulata x C. maxima)*	*C. sinensis*	Navel	N	[27]	Typical non-pigmented orange							
			Vaniglia sanguigno	VS	[27]	Lycopene-rich and acidless flesh						*	
			Tarocco Lempso	TL	[27]	Highly pigmented in the rind and less in the flesh							
			Tarocco Ippolito	TI	[27]	Highly purple, internally and externally							
			Doppio sanguigno	DS	[27]	Rind intensely red-coloured at fully maturity; flesh well-coloured							
			Moro	M	[27]	Highly pigmented, internally and externally							
	*C. reticulata x C. sinensis*	Mandarin-like	Sunred	S	[28,29]	Seedy fruits containing three-times more anthocyanins than Moro							
**Citron & its direct hybrids**	*C. medica*	*C. medica*	Diamante	CD	[30]	Typical citron							
			Buddha’s hand	MB	[30]	Fingered citron, purple flower bud and purple-tinted open flowers							
	*C. medica* hybrid	Unknown parent	Incomparabile lemon	LI	[31]	Ancient origin described as hybrid between sour orange and citron							
	*C. reticulata x C. medica*	*C. limonia*	Volkamer lemon	CV	[30]	Used as rootstock							
			Rangpur lime	RaL	[30]	New shoots lightly purple-tinted; flowers petals deeply purple-tinted							
			Red Lime	RL	[30]	New shoots lightly purple-tinted; flowers petals deeply purple-tinted							
			Lima rossa corrugata	LR	[30]	New shoots lightly purple-tinted; flowers petals deeply purple-tinted							
	*C. aurantium x C. medica*	*C. limon*	Zagara bianca	ZB	[30]	Non-pigmented flowers and young leaves							
			Femminello Adamo	FA	[30]	Typical lemon							
			Pink fleshed	PF	[30]	Also called ‘Variegated Pink Fleshed Eureka Lemon’; rind variegated in green and yellow, fading during the ripening; lycopene-rich flesh						*	*
	*C. micrantha x C. medica*	*C. aurantifolia*	Mexican lime (tornless)	LMT	[30]	Also called ’West Indian lime’; new shoots, flower buds and young flowers faintly purple-tinted							
		*C. celebica*	Southwickù CRC2453	CC	[32]	Papedocitrus							
**Other *Citrus* species**	*(C. maxima x C. reticulata) x C. medica*	*C. meyeri*	Meyer lemon	LM	[30]	Flowers and new shoot purple-tinted							
	*C. limon x C. aurantifolia* (3n)	*C. latifolia*	Tahiti lime	LT	[30]	Triploid; purple coloration usually faint and evanescent in both flowers and shoots							
**Subgenus *Papeda* and other genera**	*Microcitrus australasica* x (*Fortunella* sp. x *Citrus* sp)	*Citrus* hybrid	Faustrime	MAF	[30]	Trigeneric hybrid							
	genus *Microcitrus*	*M. australasica*	Sanguinea	MAS	[30]	Red-pulped variety of the Australian finger-lime							
	genus *Murraya*	*M. paniculata*	-	MP	[30]	Australian origin; ornamental uses; orange-coloured berries							*
	genus *Severinia*	*S. disticha*	-	SD	[30]	Primitive Citrus, also called ’Philippine Box Orange’; yellowish-green peel at maturity (#)							
		*S. buxifolia*	-	SB	[30]	Primitive Citrus, also called ’Chinese box orange’; black berries at maturity							
	subgenus *Papeda*	*C. hystrix*	-	CH	[30]	Typical “double” leaves with wide petioles; petals yellowish white or red-tinted							
		*C. latipes*	-	CL	[32]	Wild nonedible citrus species; commonly called “Khasi papeda”							

**Table 2:** Characteristics of primers used for qRT-PCR analysis. Asterisks indicate that primers had been used previously (* [25]; ** [12]; *** [19]).

Gene Classification	Gene	Gene Position	Sequence 5′ 3′	Fw/Rev	Amplicon (pb)
**Housekeeping**	EF *	Cs8g16990	AAGCTGGTATCTCCAAGGATGGT	Fw	72
			CCAAGGGTGAAAGCAAGCAA	Rev	
**Phenylpropanoid pathway**	PAL	Cs6g11950	GGAAGCTCATGTTTGCCCAA	Fw	118
			TCAGCGCCCTTGAAACCATA	Rev	
**Early biosynthetic genes**	CHS	Cs2g14720	CCAGGCTGATTATCCCGACT	Fw	90
			TTGTCACACATGCGCTTGAA	Rev	
	CHI	Cs7g28130	TCCAGGATCAACAAAGTCGCA	Fw	95
			ACACTCCTATCGCCGTGAAC	Rev	
	F3H	Cs1g25280	ATGGCTCCTTCAACCCTCAC	Fw	86
			ACCTTGGGACGCTCATCTTG	Rev	
**Late biosynthetic genes**	DFR	Cs3g25090	TGCGTGGAAGTTTGCTGAAG	Fw	101
			TGAGACTGGGTGGCATTGAC	Rev	
	ANS	Cs5g09970	CACTTGGCTTGGGACTGGAA	Fw	114
			CCAGTTCTGGTTGAGGGCAT	Rev	
	UFGT	Cs5g24820	TGATCGGGAGGCCATTCTTT	Fw	99
			TGCAAATCCCTCCACCATCT	Rev	
**Anthocyanins vacuolization**	GST	Cs6g15900	GGGACAGCTTCACATTGGC	Fw	73
			CCATTCCAGCTTCGTTCAT	Rev	
**Transcription factors**	CsRuby1 **	Cs6g17570	AGCTGCTGGGCAACAGATGGT	Fw	68
			CTTCACATCGTTCGCTGTTC	Rev	
	Noemi ***	Cs5g31400	CAGGAACCGGTTATGATAGGTAGC	Fw	80
			TCTGGCGTCAATTCTTCTTCCGGTG	Rev	

## Supplementary Materials

The following are available online at https://www.mdpi.com/2073-4425/11/7/807/s1, Figure S1. Geo-metereological conditions are illustrated as plots representing weather indexes from SIAS database for Riposto (Acireale—(37°62′ N, 15°16′ E, 102 m a.s.l.) and Lentini (Palazzelli—37°20′ N, 14°53′ E, 48 m a.s.l.) sites. The smoothed line was obtained by Loess regression approach. Fill color represents the site, the line color represents the site and year combination. (a) Daily mean temperature (Tm); (b) Daily min temperature (Tmin); (c) Daily max temperature (Tmax); (d) Daily Relative humidity as max value (RH); (e) Daily Relative humidity as min value (RH); (f) Potential evapotranspiration (mm); (g) Precipitation (mm) as monthly amount. The soil composition for accessions sampled in Acireale consists of sand 94%, loam 2%, clay 4%, pH 7.2, active lime 0%, organic matter 0.58%, available P Olsen 9 mg kg^−1^. The soil composition for sweet oranges and Sunred hybrid sampled in Lentini is reported in [18]. Figure S2. ClustalW alignment (CLC Sequence Viewer software) of amplicons for each gene is shown. (a) PAL and Early Biosynthetic Genes (CHS, CHI, F3H), (b) Late biosynthetic genes (DFR, ANS, UFGT) and GST, (c) transcription factors (Ruby and Noemi). The species are: *Citrus sinensis*, *C. medica*, *Severinia buxifolia* and *Murraya paniculata* include accessions collected and analyzed. *C. maxima* and *C. reticulata* represent one of the parents of species considered in the study. *C. ichangensis* and *Fortunella hindsii* have been considered because they are among the species for which the full genome is publicly available and for which the sequence can be used to support the conservation of genes evaluated for expression analysis. Figure S3. Flow diagram of the image analysis. (A) the picture of petals on green background; (B) the binary mask of petals obtained with SIOX segmentator; (C) output scheme for analyzing particles which needs image A and mask B to do the image analysis; D) output Table for analyzing particles, the ID corresponding to the petals ID plotted in the scheme C. Figure S4. Color of style of a flower of *Femminello Siracusano* 2kr lemon with (a) non regular and (b) normal development. Figure S5. Flowers showing the color of the stigma. The style is pigmented in (a) ‘Sunred’ and (b) ‘Moro’, not pigmented in (c) ‘Doppio sanguigno’, (d) ‘Tarocco’ or (e) ‘Navel’. (a), (b), (c) and (d) are blood oranges, (e) represents a common blond orange. Table S1. Data showing the homology of the amplicons for each gene. Concerning the species, *Citrus sinensis*, *C. medica*, *Severinia buxifolia* and *Murraya paniculata* include accessions collected and analyzed here. *C. maxima* and *C. reticulata* represent one of the parents of species considered in the study. *C. ichangensis* and *Fortunella hindsii* have been considered because they are among species for which the full genome is publicly available and for which sequence can support the conservation of genes evaluated for expression analysis. The number of different nucleotides and the correspondent modification in the amino acid in the primers are reported. Table S2. Anthocyanin and lycopene quantification in the rind of *Murraya paniculata* and in the flesh of ‘Navel’, ‘Tarocco Lempso’, ‘Tarocco Ippolito’ and ‘Moro’ oranges. Table S3. Significant values of Pearson’s correlation coefficient among gene expressions reported for each tissue. Table S4. Weights and % of explained variance of scaled gene expression of the four components of the PLS model for prediction of petal color intensity. Table S5. Paired test of qualitative presence/absence of anthocyanin pigmentation in petals, rind, flesh/juice, and stigma. Student *t*-test of normally distributed values of gene expression and Wilcox test for non-normal distributed values of gene expression. Only tissues with at least one significant value are reported. The significant values are shown in bold.
