# Supplementary figures and images for: Target-Genes Reveal Species and Genotypic Specificity of Anthocyanin Pigmentation in Citrus and Related Genera
Source: Genes (Basel). 2020 Jul 16;11(7):807. doi: 10.3390/genes11070807 (PMC7397085; doi:10.3390/genes11070807)

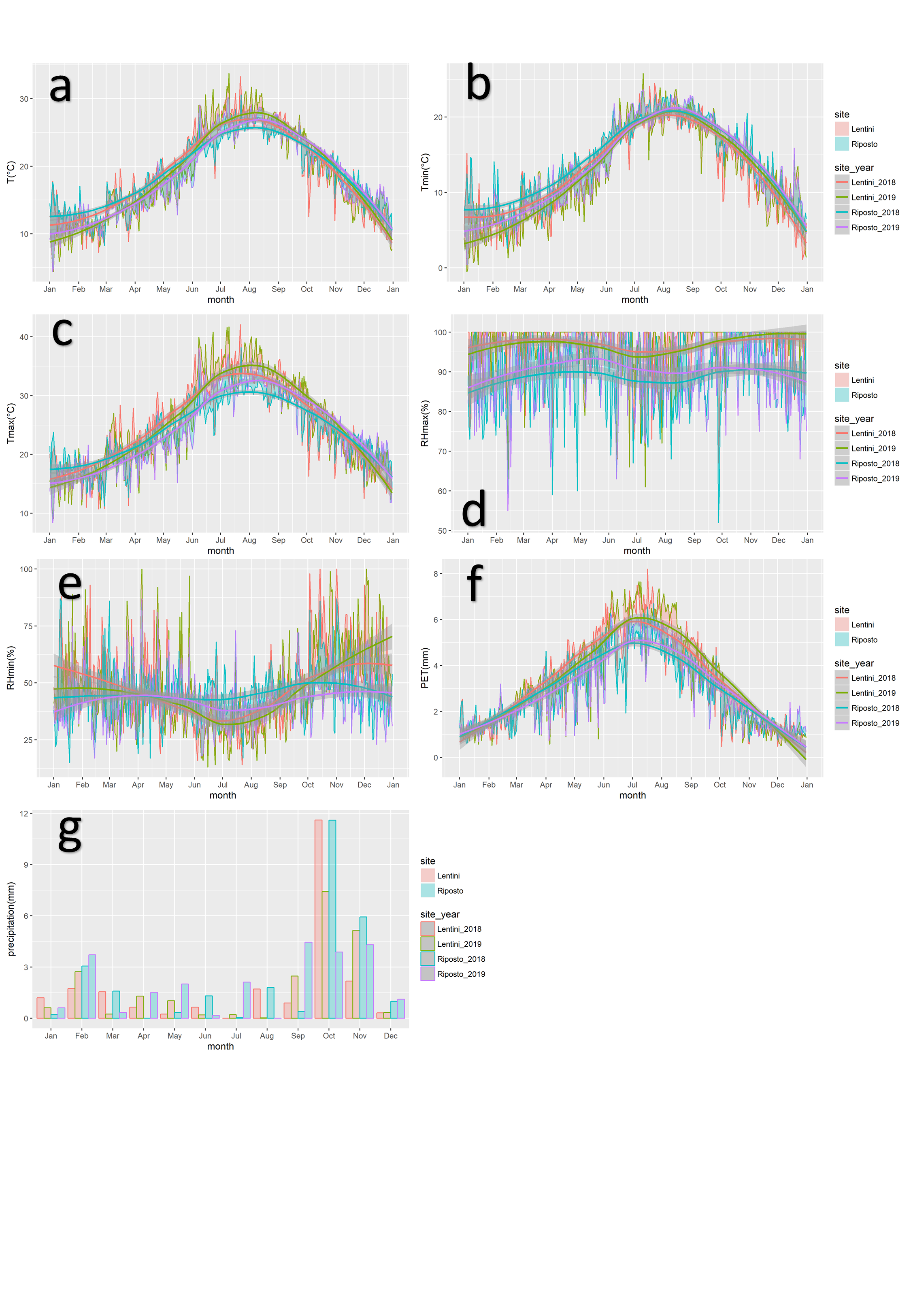

Supplement: Supplementary file 1 [file genes-11-00807-s001.zip › Figure S1.tif]

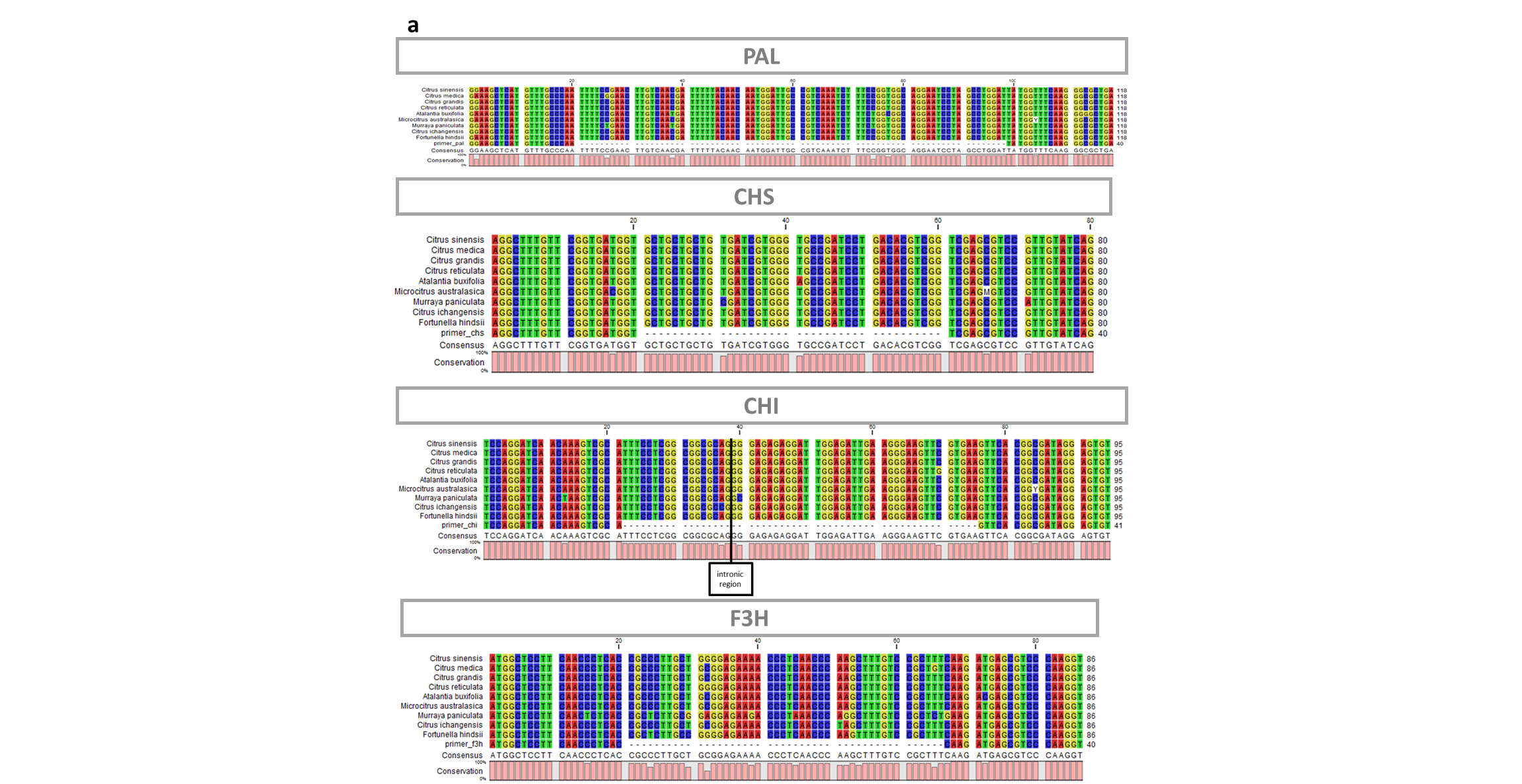

Supplement: Supplementary file 1 [file genes-11-00807-s001.zip › Figure S2A.tiff]

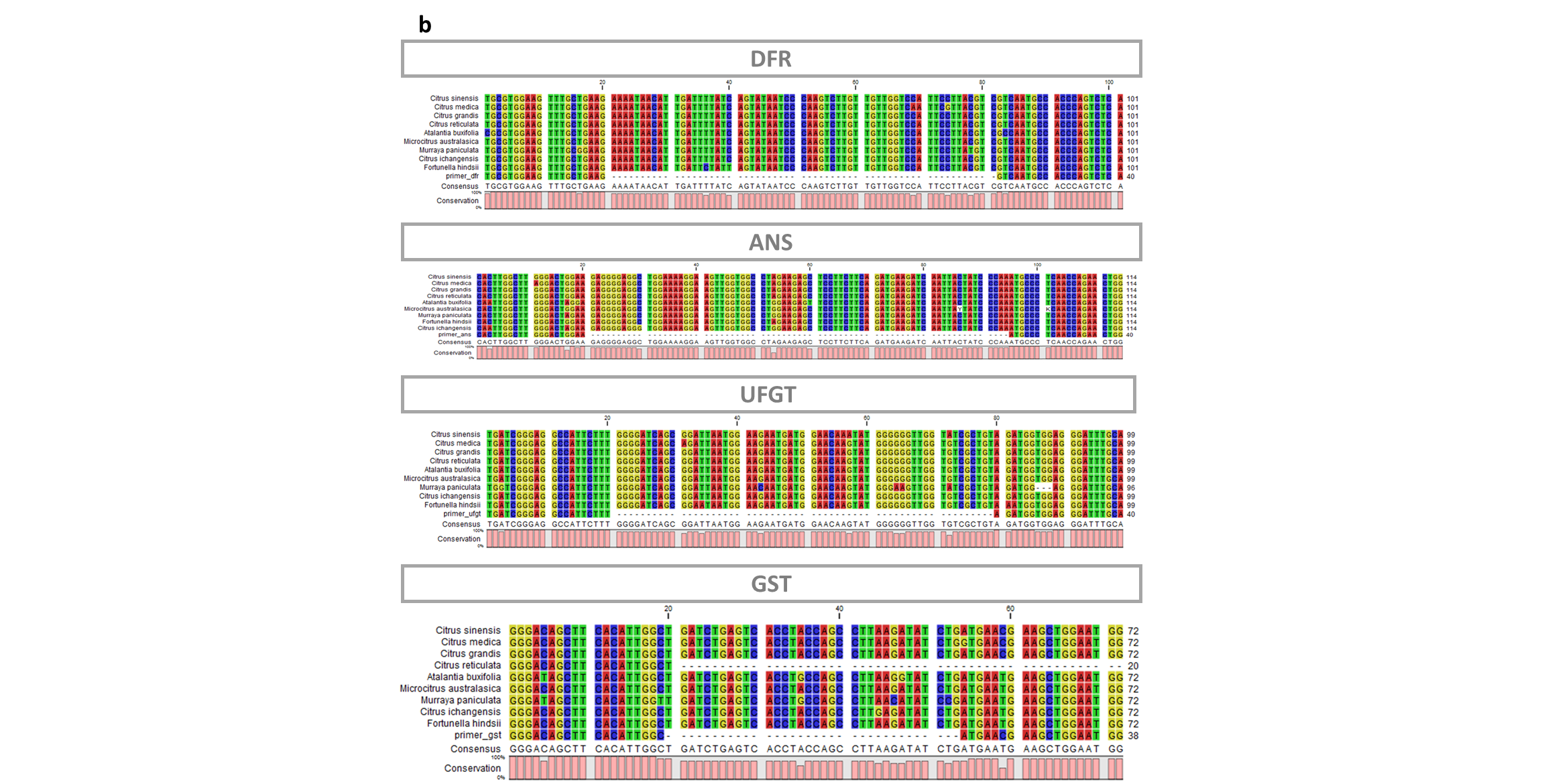

Supplement: Supplementary file 1 [file genes-11-00807-s001.zip › Figure S2B.tiff]

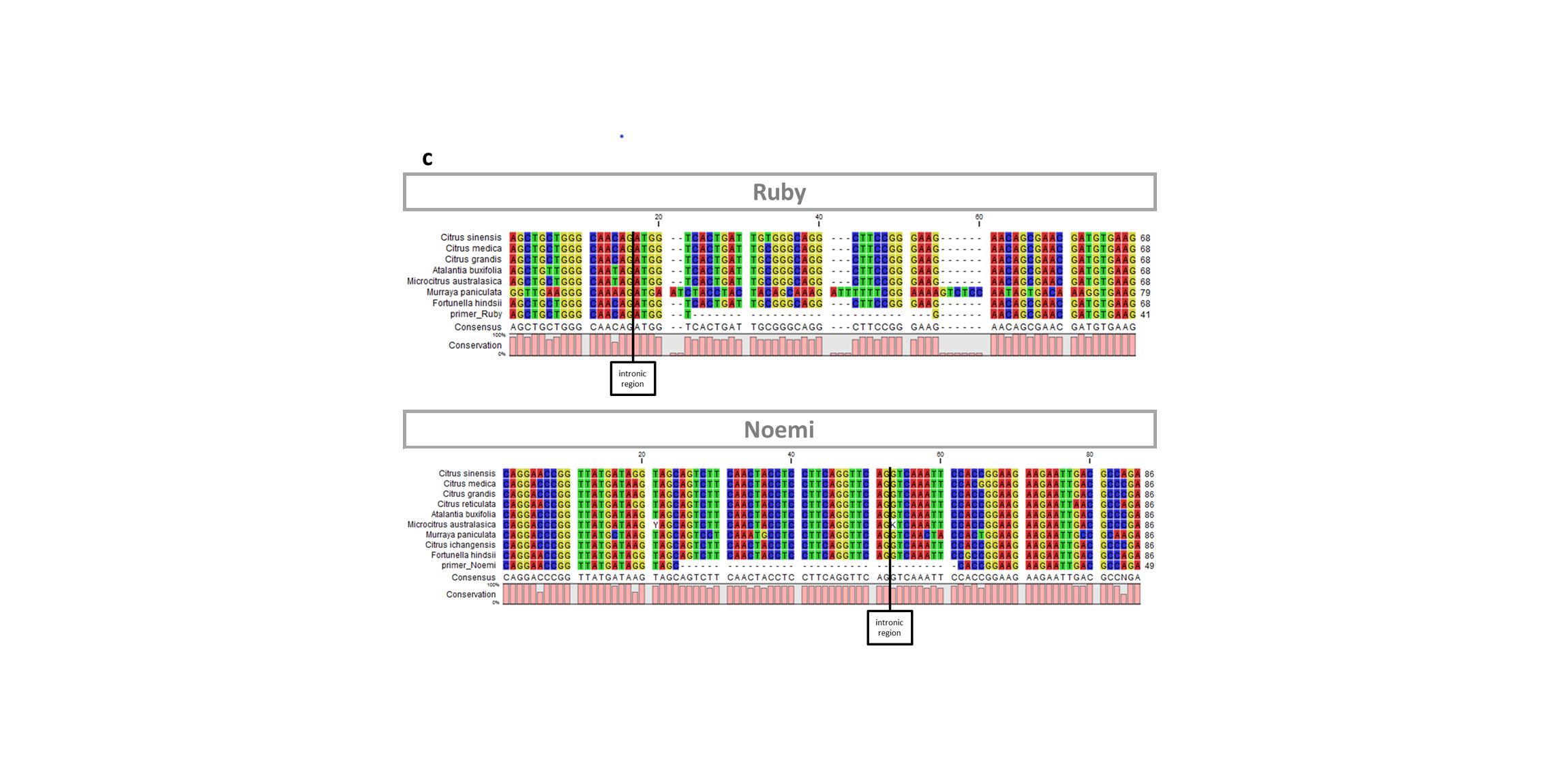

Supplement: Supplementary file 1 [file genes-11-00807-s001.zip › Figure S2C.tiff]

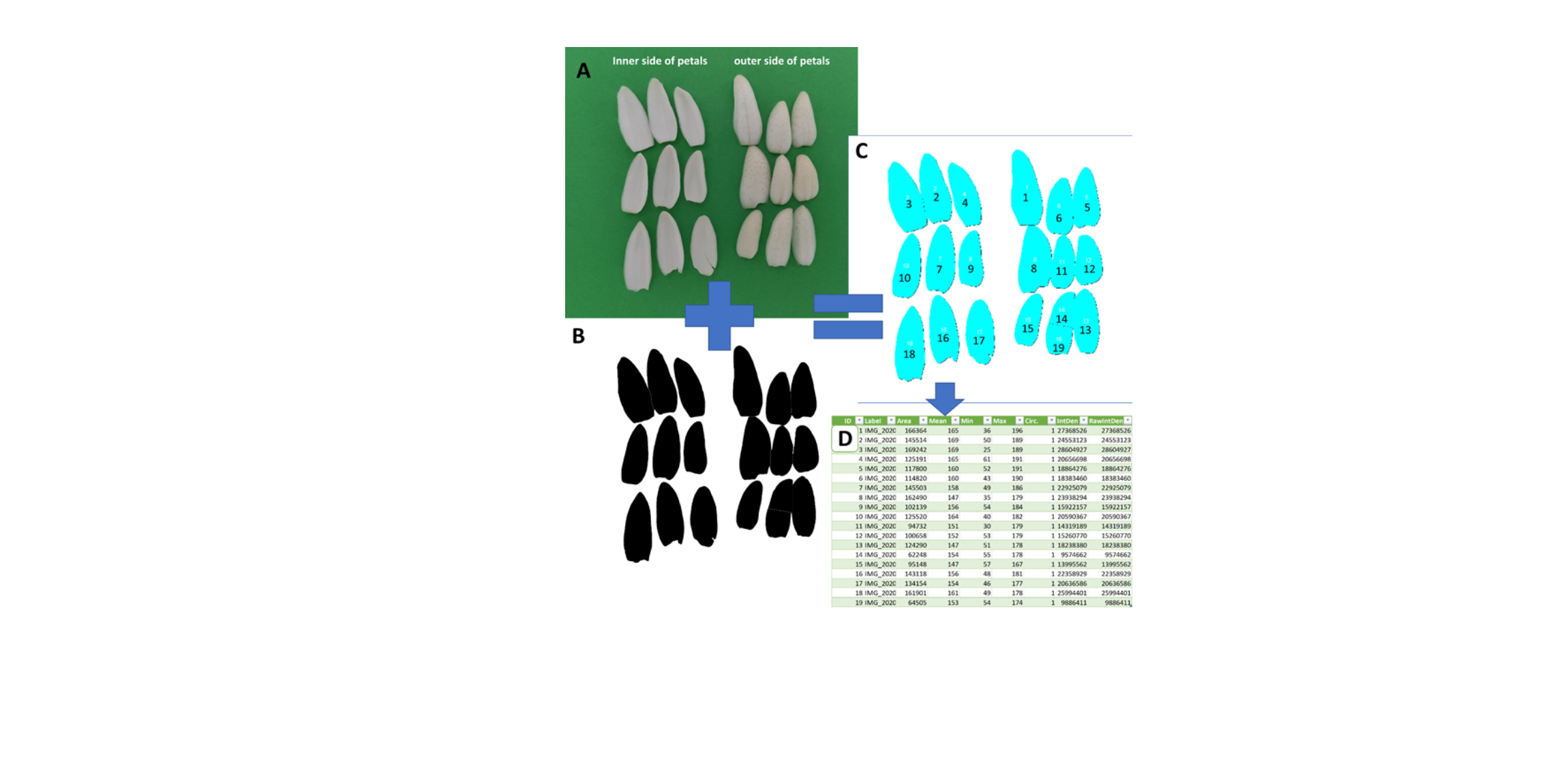

Supplement: Supplementary file 1 [file genes-11-00807-s001.zip › Figure S3.tiff]

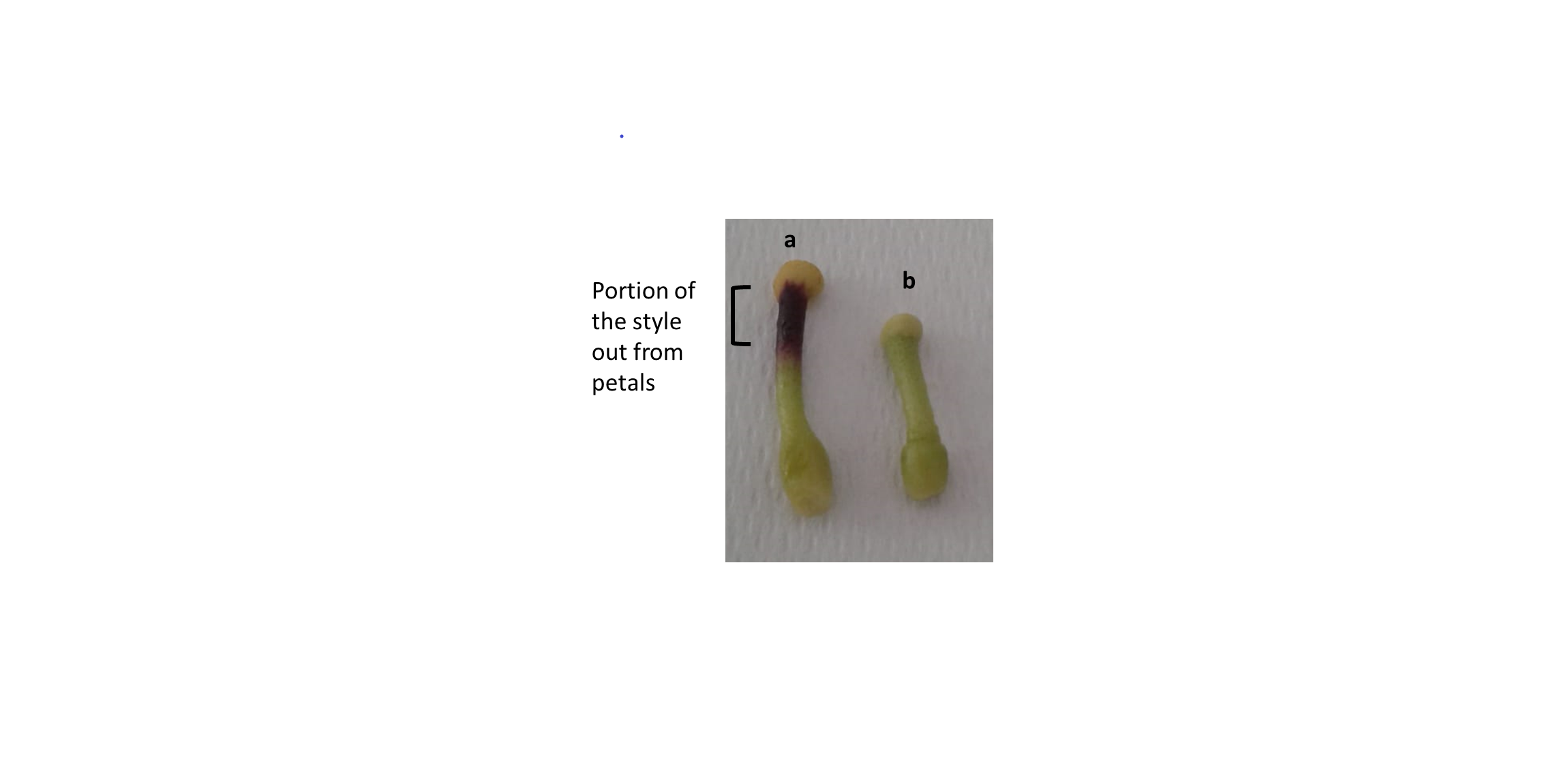

Supplement: Supplementary file 1 [file genes-11-00807-s001.zip › Figure S4.tiff]

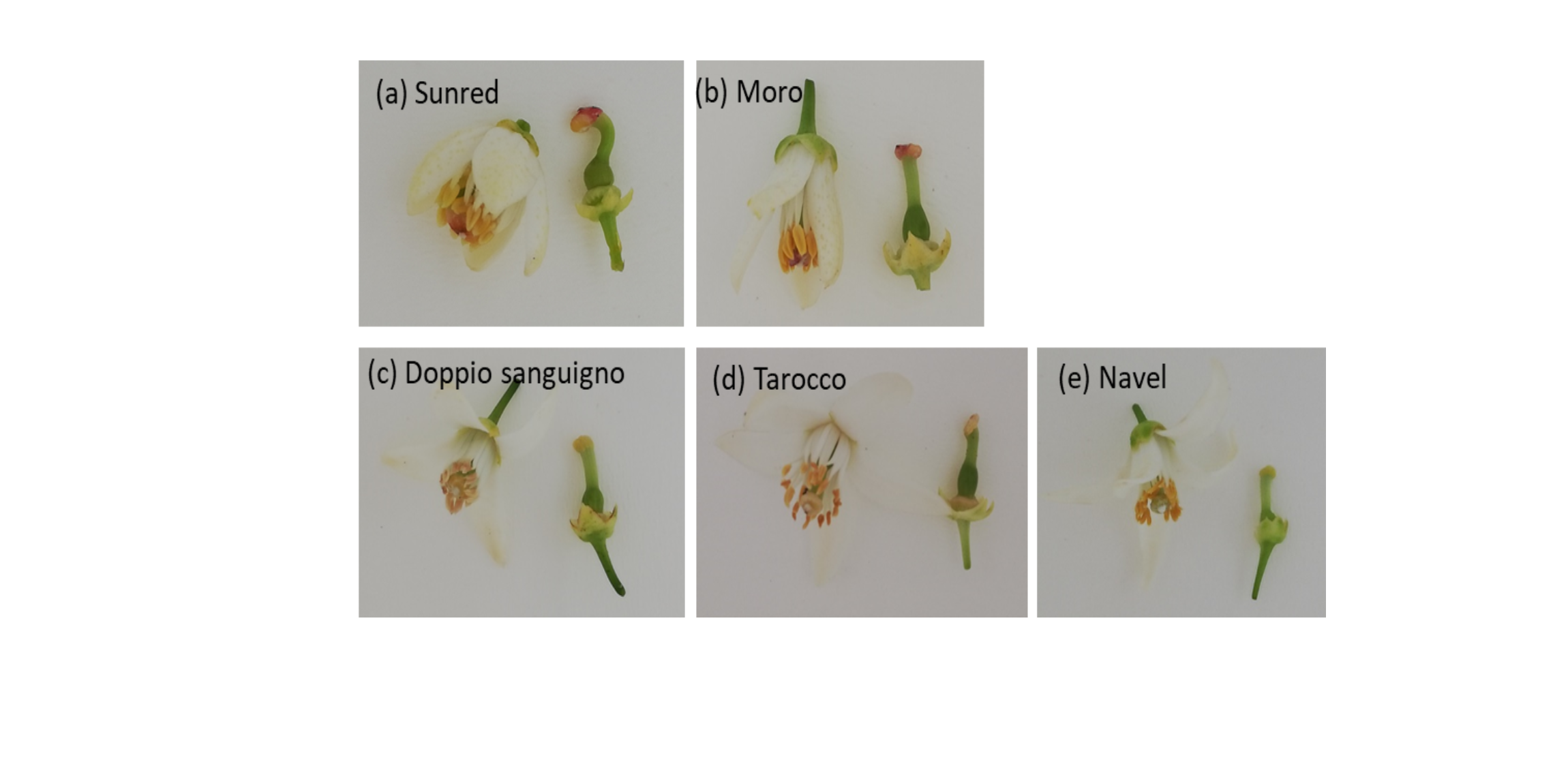

Supplement: Supplementary file 1 [file genes-11-00807-s001.zip › Figure S5.tiff]
